# Bacterial social interactions drive the emergence of differential spatial colony structures

**DOI:** 10.1186/s12918-015-0188-5

**Published:** 2015-09-16

**Authors:** Andrew E. Blanchard, Ting Lu

**Affiliations:** Department of Physics, University of Illinois at Urbana-Champaign, 1110 West Green Street, Urbana, 61801 USA; Department of Bioengineering, University of Illinois at Urbana-Champaign, 1304 West Springfield Avenue, Urbana, 61801 USA; Institute for Genomic Biology, University of Illinois at Urbana-Champaign, 1206 West Gregory Drive, Urbana, 61801 USA

**Keywords:** Community structures, Social interactions, Bacterial colonies, Spatiotemporal patterns

## Abstract

**Background:**

Social interactions have been increasingly recognized as one of the major factors that contribute to the dynamics and function of bacterial communities. To understand their functional roles and enable the design of robust synthetic consortia, one fundamental step is to determine the relationship between the social interactions of individuals and the spatiotemporal structures of communities.

**Results:**

We present a systematic computational survey on this relationship for two-species communities by developing and utilizing a hybrid computational framework that combines discrete element techniques with reaction-diffusion equations. We found that deleterious interactions cause an increased variance in relative abundance, a drastic decrease in surviving lineages, and a rough expanding front. In contrast, beneficial interactions contribute to a reduced variance in relative abundance, an enhancement in lineage number, and a smooth expanding front. We also found that mutualism promotes spatial homogeneity and population robustness while competition increases spatial segregation and population fluctuations. To examine the generality of these findings, a large set of initial conditions with varying density and species abundance was tested and analyzed. In addition, a simplified mathematical model was developed to provide an analytical interpretation of the findings.

**Conclusions:**

This work advances our fundamental understanding of bacterial social interactions and population structures and, simultaneously, benefits synthetic biology for facilitated engineering of artificial microbial consortia.

**Electronic supplementary material:**

The online version of this article (doi:10.1186/s12918-015-0188-5) contains supplementary material, which is available to authorized users.

## Background

**Fig. 1:**
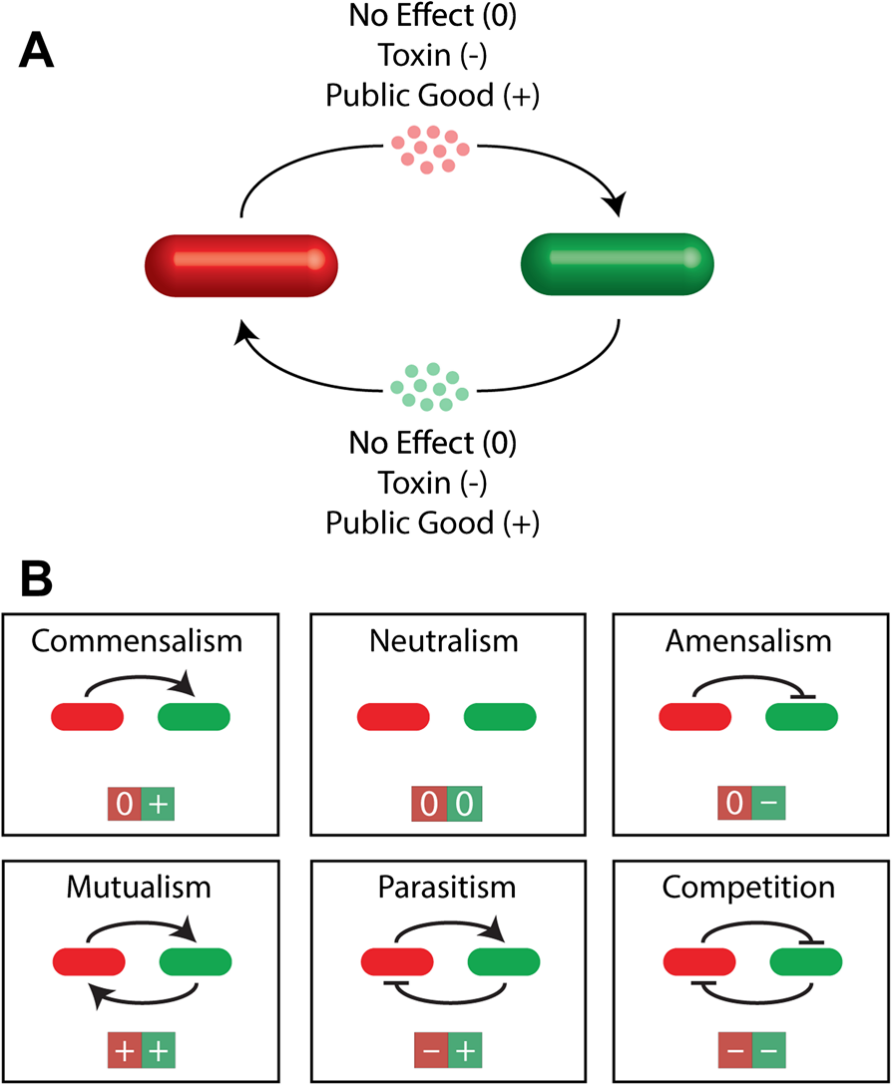
Pairwise social interactions in bacteria. **a** Cell interactions implemented through the production of diffusible chemicals. The chemicals from one species may be deleterious (e.g, toxin), beneficial (e.g., public good), or neutral to the growth of the other. **b** Six distinct types of social interactions in a two-species population, including commensalism, neutralism (control), amensalism, mutualism, parasitism, and competition

Bacteria are single-celled organisms but are highly social when they live in natural environments. They interact with each other in different habitats, across different species, and also through different modes [1–5], thereby generating a stunningly wide spectrum of social behaviors from cooperation and communication to synchronization [3, 6, 7]. For instance, even for a simple ecosystem consisting of only two species (Fig. 1a), there are six possible distinct types of interaction (Fig. 1b) including neutralism, commensalism, amensalism, competition, mutualism, and parasitism [8].

The social interactions of bacteria alter the physiology, gene expression, and survival of individual cells and also enable the collective behaviors of populations, therefore significantly impacting the dynamics and functionality of an entire community. For instance, through cooperation–one of the major forms of cellular interactions, bacteria can achieve diverse goals: Pseudomonas bacteria cooperate to form biofilms to shed planktonic, disperser cells into the water under a diurnal rhythm [9]; Salmonella releases virulence factors collectively upon reaching a threshold density [3]; and Myxobacteria form fruiting bodies to protect from attack while facilitating dispersal [10]. Similarly, bacteria also acquire benefits via competition–another common mode of interaction. For example, *Lactobacillus salivarius* exerts positive effects on host health by producing a bacteriocin *in vivo* against the invasive foodborne pathogen *Listeria monocytogenes* [4], *Pseudomonas fluorescens* mutants overproduce extracellular polysaccharide to gain enhanced accessibility to oxygen [1, 11], and *Burkholderia thailandensis* mediates their own biofilm formation by excluding competing species through contact-dependent inhibition [12]. These complex and intriguing phenomena enabled by cellular interactions motivate us to ask the following question: How do bacterial social interactions impact the functionality of an entire community?

To answer this question, one fundamental step is to determine the relationship between the social interactions of individuals and the spatiotemporal structures of communities. The underlying reason is that the ability of a community to perform a specific function relies upon the collective behaviors of individual cells with a given spatiotemporal arrangement and a corresponding relative abundance. Additionally, understanding the cellular interaction-community structure relationship will be instrumental to social evolution theory in the context of microbes [2, 6, 7, 13, 14]. It will also advance the understanding of disease pathogenesis as well as the development of better treatment strategies. Moreover, it will offer invaluable knowledge for the design and construction of engineered microbial consortia for desired functionality, thereby advancing synthetic biology for community-based gene circuit engineering.

Recently, there has been considerable interest in understanding the relationship between bacterial community structure and individual social interactions. For instance, researchers have attempted to understand the emergence of spatial segregation in expanding microbial colonies [15] and the patch length scale of mutualistic species [16]. Additionally, a variety of engineered microbial ecosystems have been developed to implement various social interactions [17–20] and further applied to study population dynamics and spatial structure [18, 21–24].

Simulations of social interactions in spatially structured populations have commonly utilized continuous partial differential equations to model both cell movement and diffusible molecules, with associated bulk diffusion constants [16, 18, 22, 24, 25]. An alternative approach is to track individual cell movements, which can be driven by lattice-based rules [26, 27] or mechanical force calculations [28–36]. In particular, incorporating force calculations has been vital to accurately modeling the true dynamics of expanding populations. However, despite the great advances made by these efforts, there has been a lack of systematic computational investigation into the social interaction-community structure relationship that incorporates both mechanical forces and diffusible chemicals.

Here, we present a systematic survey on the relationship between the spatial structure of bacterial communities and the social interactions of individuals. We first develop a hybrid computational framework for modeling bacterial communities that combines discrete element techniques for force calculations with reaction-diffusion equations. We then employ the framework to simulate the structure of growing colonies with different pairwise interactions utilizing a two-species model system. A statistical investigation of the resulting community patterns follows, with key metrics including species abundance, colony morphology, and number of surviving lineages. To examine the generality of our findings, various initial conditions are tested for community simulation and analysis. Furthermore, we construct an ordinary differential equation model for an analytical interpretation of our findings.

## Results and discussion

### A computational framework for modeling bacterial communities

When considering a bacterial community, there are two primary classes of cellular events, namely growth dynamics (cell elongation, division, and movement) and intercellular chemical interactions (e.g. competition or cooperation). We therefore have constructed a computational framework that incorporates both classes of events to systematically explore the link between social interactions at the single cell level and population structures.

**Fig. 2:**
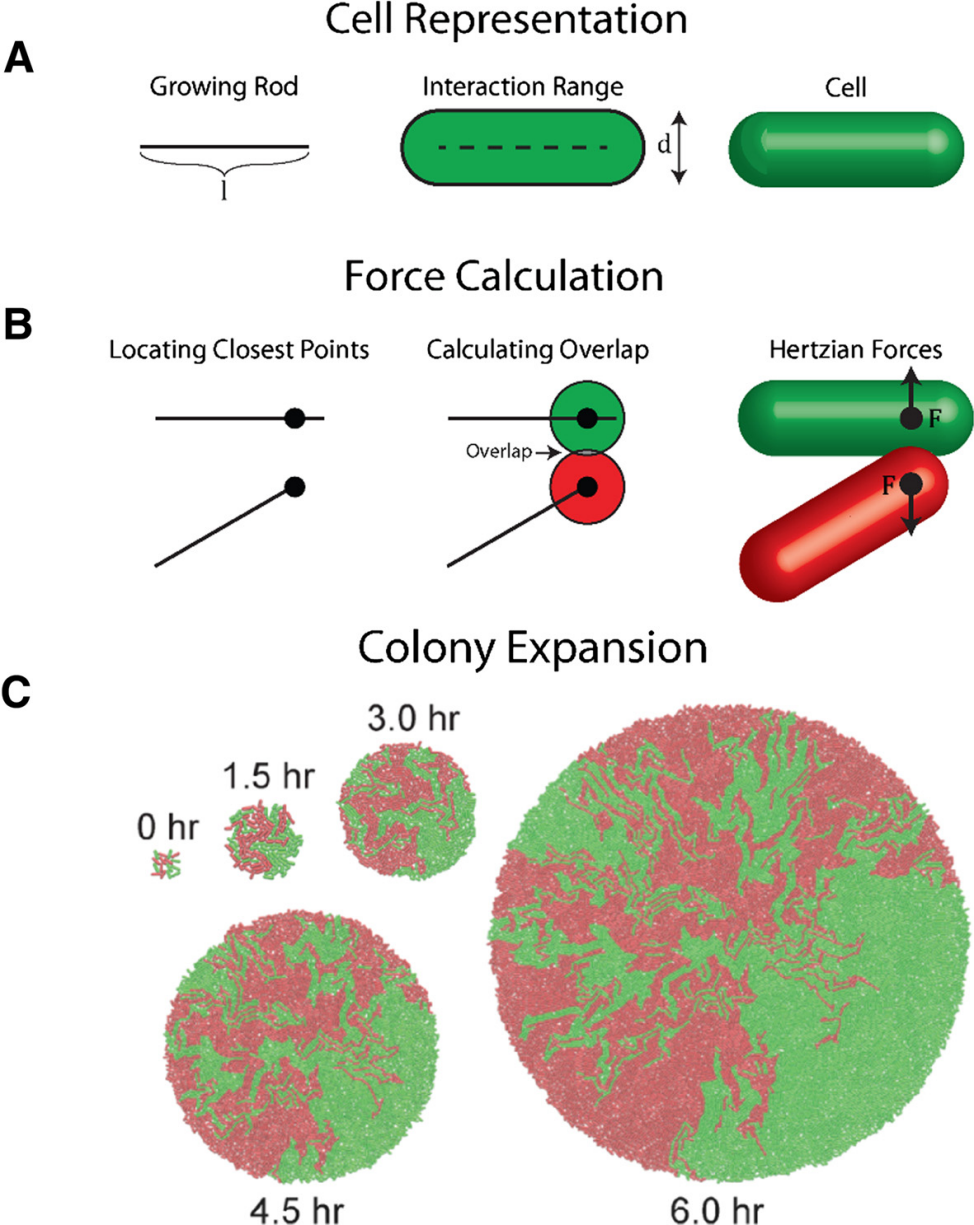
Mechanical modeling of bacterial growth dynamics. **a** Representation of individual cells. Each cell is modeled as a growing rod surrounded by a deformable elastic shell. **b** Calculation of the mechanical forces between two contacting cells. The procedure includes finding the closest points between the cells, computing the overlap between the deformable spheres at the closest points, and then calculating the corresponding Hertzian force. **c** Snapshots of a representative simulation of two-species bacterial colony expansion

To describe growth dynamics, a single bacterium (e.g. *E. coli*) was modeled as a rigid rod surrounded by a deformable shell with defined elastic properties (Fig. 2) and movement in two-dimensional space, similar to previous modeling work [28–35]. Therefore, cellular growth (i.e. elongation) can be described by increasing the rod length with a rate determined by the local availability of nutrients and chemicals; cellular division can be mimicked by dividing the rod into two once its length reaches a threshold. To model cellular movement, a discrete element technique [31, 33, 37] was employed to describe the mechanical forces generated by spatial volume overlaps due to cellular growth and division (Fig. 2b). By incorporating cellular growth, division, and movement, the framework is able to successfully mimic the spatiotemporal dynamics of growing populations. Figure 2c shows time snapshots of colony expansion for a two-species population simulated using the framework.

To model intercellular chemical interactions, we classified them into two types, asocial and social. Asocial interactions correspond to the coupling of cells with the environment via the consumption of shared nutrition. In contrast, social interactions require the production and sensing of diffusible chemicals such as toxins, public goods, and signaling molecules. Notably, although the framework can be easily adapted to incorporate interactions through direct contact (e.g., contact-dependent growth inhibition) [25, 38], in this study we primarily focus on chemical interactions mediated by diffusible molecules. Thus, in the model direct contact does not affect cell growth but is responsible for generating cell movement. Reaction-diffusion equations were adopted to describe the dynamic spatial distribution of diffusible chemicals (including nutrition) as well as their interactions with cells in space. With this approach, different interaction types can be implemented by specifying the coupling between local chemical concentration and cellular growth rate. By incorporating reaction-diffusion equations and a discrete element based description of growth dynamics, the computational framework captures realistic features of interacting bacterial communities.

### Systematic survey of the roles of cellular interactions in determining community structure

To examine how social interactions impact community structure, we employed the above computational framework to systematically survey spatiotemporal patterns emerging from communities with different cellular interactions. Here, the colony expansion of a two-species bacterial population was used as a model system because it retains many key features of complex communities, such as spatial expansion, nutrient shielding, and both mechanical and chemical interactions.

**Fig. 3:**
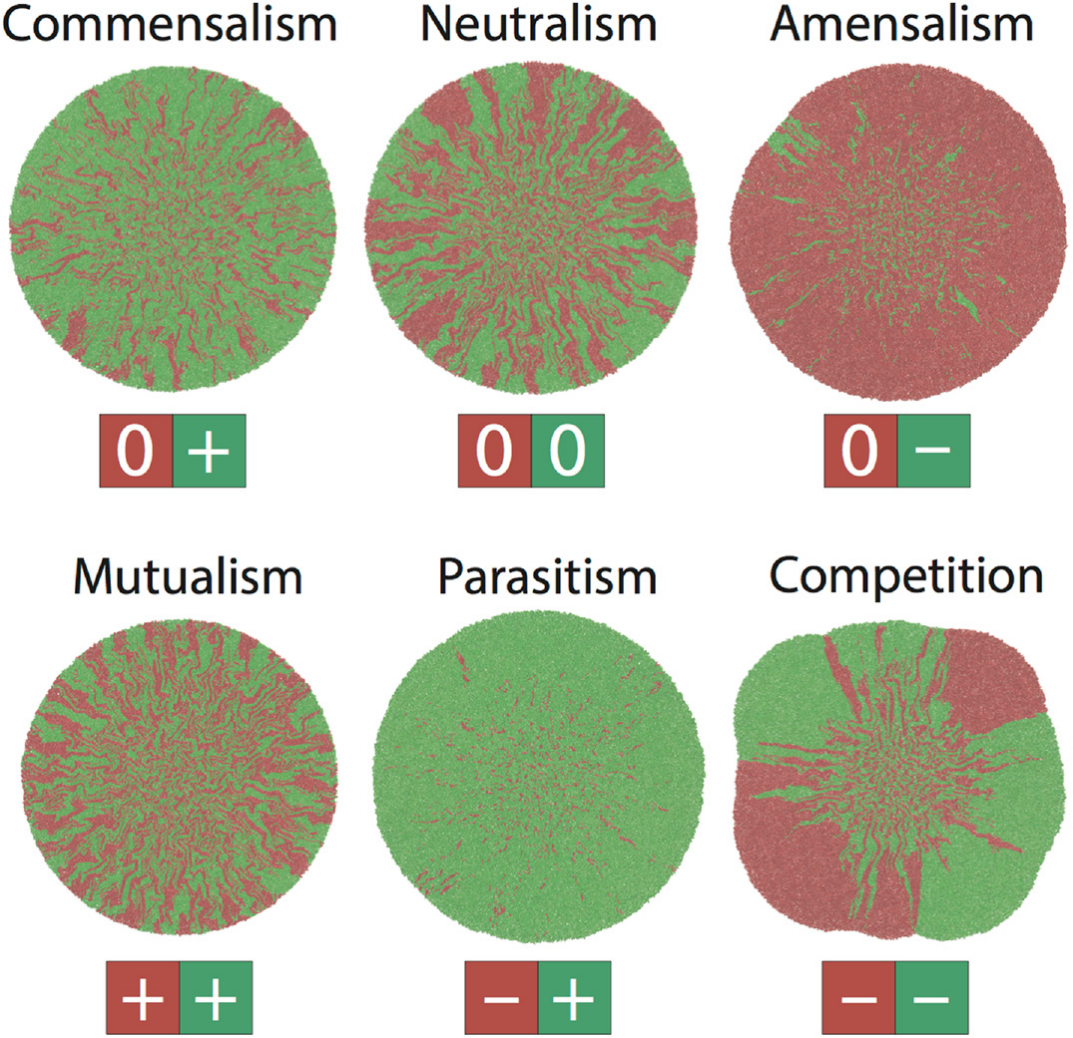
Representative spatial structures emerged from two-species bacterial communities with different social interactions

Figure 3 shows the community structures of bacterial populations for all possible distinct pairwise interactions, including commensalism, neutralism (control), amensalism, mutualism, parasitism, and competition as listed in Fig. 1b. For simplicity, a fixed initial cell density, an equal relative abundance, and a well-mixed spatial distribution (See “Simulation protocols” in “Methods”) were used for all of the simulated communities. In addition, all parameters governing growth, division, and nutrient consumption were the same for both the green and the red species; only the parameters relating to social interactions were varied (Detailed parameters are available in Supporting Information (Additional file 1: Section 1). Clearly, different social interactions resulted in qualitatively different population structures for expanding colonies.

Interestingly, communities with asymmetrical social interactions (commensalism, amensalism, and parasitism) have an unbalanced structure where the species benefiting from social interactions dominates the population, such as the green cells in the commensal and parasitic populations. Accordingly, the species hurt by social interactions becomes the minority of a community or even dies out, including the green cells for amensalism and the red cells in parasitic populations. In contrast, communities with symmetrical interactions (mutualism, neutralism, and competition) have roughly equal abundances for the two species regardless of their interaction types. However, the spatial characteristics of these communities are distinct: The mutualistic community has a higher degree of spatial homogeneity (green-red mixing) compared with control, consistent with a recent experimental report [16]; on the contrary, in the competing community, the two species display a high degree of spatial segregation.

To obtain a statistical understanding of the above findings, we decided to perform multiple runs for each of the ecosystems. In addition to the spatially well-mixed initial condition, we utilized a set of random initial conditions. Maintaining an equal abundance and a fixed density, cells were placed on a spatial grid with random orientation, length, and species type. Furthermore, to achieve a quantitative statistical description of the differential structure characteristics, we utilized a set of metrics to quantify the outcomes of the simulations, including relative abundance (e.g., fraction of green cells), colony roughness, number of surviving cell lineages, and colony sectors (see “Quantitative metrics” in “Methods”).

**Fig. 4:**
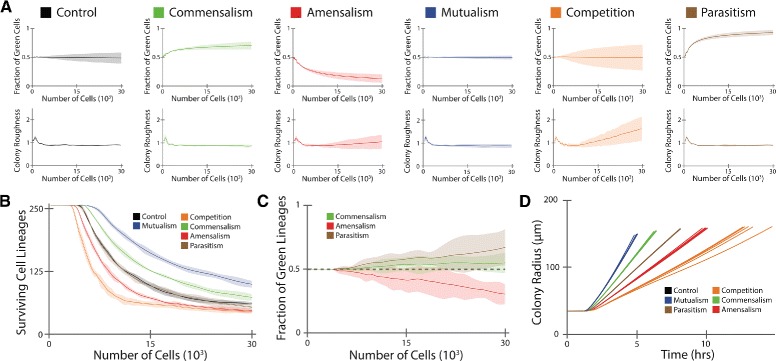
Statistical analysis of the role of social interactions in determining community structure. **a** Relative species abundance (the fraction of green cells) and colony roughness as functions of total cells in the population. The solid lines correspond to the mean values, and the shaded regions reflect two standard deviations. The statistics were obtained from six runs of simulations. **b** The number of surviving lineages as a function of total cells. Communities with deleterious interactions (amensalism and competition) have a faster decay of lineage number compared with control (neutralism), in contrast, those with beneficial interactions show a slower lineage decay. Control and parasitism have nearly indistinguishable plots. **c** Fraction of green lineages in total lineages. **d** Colony radius as a function of time for all 36 simulations runs. Differential colony expansion rates were observed for communities with different interactions. Control and parasitism have nearly indistinguishable radial expansion due to similar overall growth rates

As shown in the top row of Fig. 4a as well as Table 1, social interactions indeed resulted in a dramatic difference for relative species abundance (fraction of green cells here). The community structures with asymmetrical interactions (commensalism, ammensalism, and parasitism) have uneven relative abundance, with the species having a growth advantage dominating the population; those from symmetrical interactions (control, mutualism, and competition) have an even species abundance on average, consistent with our qualitative findings above. More interestingly, we found that, among the three communities with symmetric interactions, the mutualistic community has the lowest variance and the competing community has the largest variance, suggesting a strong correlation between population robustness and the type of social interaction.

**Table 1:** Statistics of the metrics for the six communities with an equal initial abundance and a high density. The mean and the standard deviation are shown

	Control	Commensalism	Amensalism	Competition	Mutualism	Parasitism
Green Fraction	0.49±0.05	0.71±0.03	0.13±0.04	0.49±0.11	0.49±0.02	0.93±0.02
Roughness	0.90±0.02	0.87±0.02	1.05±0.15	1.60±0.26	0.87±0.04	0.91±0.02
Lineages	61.17±1.33	73.33±3.33	46.67±1.97	44.83±1.72	99.50±3.56	54.00±2.45
Green Lineages	28.50±4.04	40.17±2.23	14.17±1.72	20.50±2.88	48.00±3.74	36.17±3.37
Sectors	24.00±5.02	20.00±5.29	3.67±1.97	7.50±2.07	32.17±8.18	1.83±1.17

Another key factor for expanding colonies is the morphology. Here, we characterized morphology by determining the spatial fluctuations of the population edge around the average radius (i.e. colony roughness). As shown in the bottom row of Fig. 4a, all of the communities showed an approximately constant mean roughness during colony growth, except for those with amensalism and competition interactions, where a linear increase in colony roughness was observed for increasing total cell number. Associated with this finding, the variances for colony roughness with amensalism and competition were also elevated over the other interaction types.

We further examined the effects of interactions on the diversity and spatial distribution of a community by measuring the surviving lineages. Here, the number of surviving lineages is defined as the number of seeding cells which have viable progeny on the expanding colony front with access to nutrients. Figure 4b shows that each interaction type confers a characteristic decay for surviving lineages. The following order is observed from lowest to highest: competition, amensalism, parasitism, control, commensalism, mutualism (Table 1). In addition to the total number of surviving lineages, we also noticed that the fraction of each species is also subject to interaction type. As shown in Fig. 4c, for the communities with asymmetric interactions, amensalism and parasitism displayed a large loss in lineages for the victim species while commensalism maintained a lineage fraction close to the control case. The lineage fraction statistics for the remaining interaction types are shown in Additional file 1: Figure S1.

As a final measure to differentiate the roles of social interactions in determining population structure, we considered the number of sectors on the expanding front of each population, where sectors were defined as spatially connected cell clusters that are greater than one hundred cells from the same species. The mean sector number provides another metric to distinguish the impacts of different interactions, with an order from lowest to highest as: parasitism, amensalism, competition, commensalism, control, mutualism (Table 1). Moreover, we noticed that the number of sectors reflects the difference between the symmetric interactions, with mutualism having the most sectors and competition having the least (Table 1).

It is important to note that, although the above statistics of colony structures have been presented as a function of total cell number for consistent comparisons, our analysis can be directly applied to time series analysis. For instance, different rates of radial expansion of the colonies can be revealed (Fig. 4d), where the differences in overall growth originate from the interaction types. Additional analysis of the community structures based on time series is available in Additional file 1: Figure S2.

Taken together, the above metrics provide a complementary characterization of the impact of social interactions on community structure. The mean species abundance can be used to classify the communities with asymmetric interactions while the variance can be applied to differentiate between symmetric interactions (Fig. 4a). The colony roughness can be employed to identify communities with deleterious interactions such as amensalism and competition (Fig. 4a). Counting surviving lineages offers an ordering for all of the interactions (Fig. 4b). Finally, sector number provides an additional metric that distinguishes between the symmetric interactions (Table 1).

From the perspective of interaction-structure relationship, deleterious interactions (amensalism and competition) cause sizable variance in relative abundance compared to the mean, a drastic decrease in surviving lineages and a rough expanding front; beneficial interactions (commensalism and mutualism), on the other hand, lead to a reduced variance in abundance compared to the mean, an enhancement in lineages, and a smooth expanding front. In addition, the communities with asymmetric interactions (commensalism, amensalism, and parasitism) have differential mean relative abundance while those with symmetrical interactions (control, mutualism, and competition) are distinct in the variance of their relative abundance. For the communities with symmetrical interactions, mutualism promotes spatial homogeneity and population robustness compared with control; in contrast, competition results in spatial segregation and population fluctuations.

Previous experimental efforts have examined the role of competition [26], neutralism [15], and cooperation [16, 26] respectively in shaping interspecies mixing in expanding colonies. Perhaps as expected, cooperation yields increased mixing over neutralism, which yields increased mixing over competition. Our simulation results reproduce this experimentally verified hierarchy, as quantified by the number of surviving lineages and colony sectors. More specifically, the control case results in the formation of sectors over time due to the expanding colony front [15]. Mutualism results in increased species mixing [16, 26], and competition results in increased segregation due to species exclusion [5, 26]. In addition, our results show that all six pairwise interaction types yield distinguishable community structures, with each metric providing a predicted order for the interaction types. The results (Fig. 4 and Table 1) provide concrete predictions concerning the quantitative effects of social interactions in natural and synthetic communities, guiding the rational design of social microbial consortia with novel functionalities.

### Dependence of community structure on initial conditions

In both natural and experimental settings, there may exist variations during initial colonization of bacterial populations in a new habitat. To examine how these variations impact our findings, we performed and analyzed a series of *in silico* colony development experiments for various initial conditions, with a primary focus on total initial cell density and relative species abundance.

**Fig. 5:**
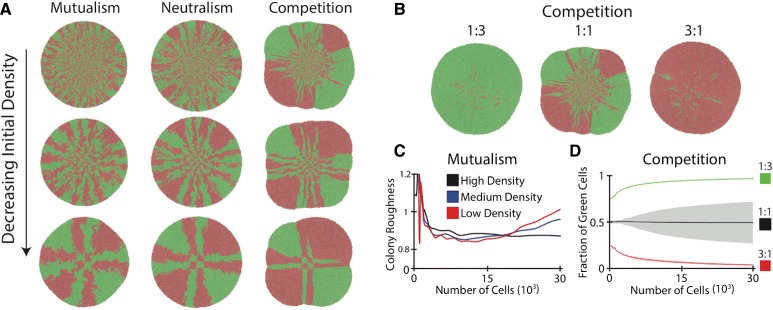
Impacts of initial conditions on community structure. **a** Representative colony patterns emerged from cells with a high, medium, and low initial density for the cases of mutualism, neutralism and competition. With the decrease of initial cell density, the general impacts of social interactions on community structure remain but their magnitudes have been reduced. **b** Representative community structures simulated from competing communities with different initial relative abundances. Alteration of the initial abundances resulted in dramatically different outcomes in competing communities. **c** Mean colony roughness of the mutualistic communities simulated in (**a**). **d** Fraction of green cells in the competing communities in (**b**)

For total initial density, we studied three representative scenarios, corresponding to high, medium, and low cell densities, while keeping an equal relative abundance. Figure 5a shows the representative colony structures of the communities with mutualism, neutralism, and competition. Additional file 1: Figures S3–S4 and Additional file 1: Tables S3–S4 show the systematic analysis of the resulting community structures using the quantitative metrics proposed in the above section. We found that, although changes in the initial conditions can alter outcomes, they typically do not destroy the characteristic features of the interaction types for a given measured quantity. For example, changes in initial density do not alter the ordering of amensalism, commensalism, and parasitism for increasing fraction of green cells reported in the previous case. In addition, the variance still increases from mutualism to control and finally to competition. The colony roughness also remains similar, with amensalism and competition displaying deviations from circular colonies (Additional file 1: Figure S5). Moreover, the ordering for the surviving lineages is also roughly preserved for decreasing density, and the deleterious interactions continue to cause decreased surviving lineages compared with beneficial interactions.

However, a smaller initial density is correlated with a smaller difference in the number of surviving lineages (Fig. 5a, Additional file 1: Figures S3–S4) and a higher colony roughness (Fig. 5c). This is because, for the same initial seeding area, a smaller initial density corresponds to a longer average cellular distance, enabling each seed cell to grow into a larger clonal aggregate before interactions play a role. For amensalism, lowering the density results in the prolonged survival of green cells (Additional file 1: Figure S6). For mutualism, lower densities result in deviations from circular colonies due to a gradient in the growth rate at the expanding front (Figs. 5a and 5c).

We also evaluated the impacts of relative species abundance by conducting simulations for different initial ratios (1:7, 1:3, 1:1, 3:1, 7:1) for a fixed total density, with the corresponding analysis shown in Additional file 1: Figures S7–S10 and Additional file 1: Tables S5–S8. The results suggest that the relative abundance in initial cells has differential influences depending on interaction type. For instance, compared to control, the communities with mutualism tend to minimize differences in the initial abundance (Additional file 1: Figure S11) while competitive communities intensify differences in the initial abundance. Figure 5b shows representative colony structures for competing communities with different initial ratios and Fig. 5d shows the evolution of relative abundance of the communities, both of which illustrate the exacerbated effect from species competition.

### Analytical interpretation of differential community structures.

**Fig. 6:**
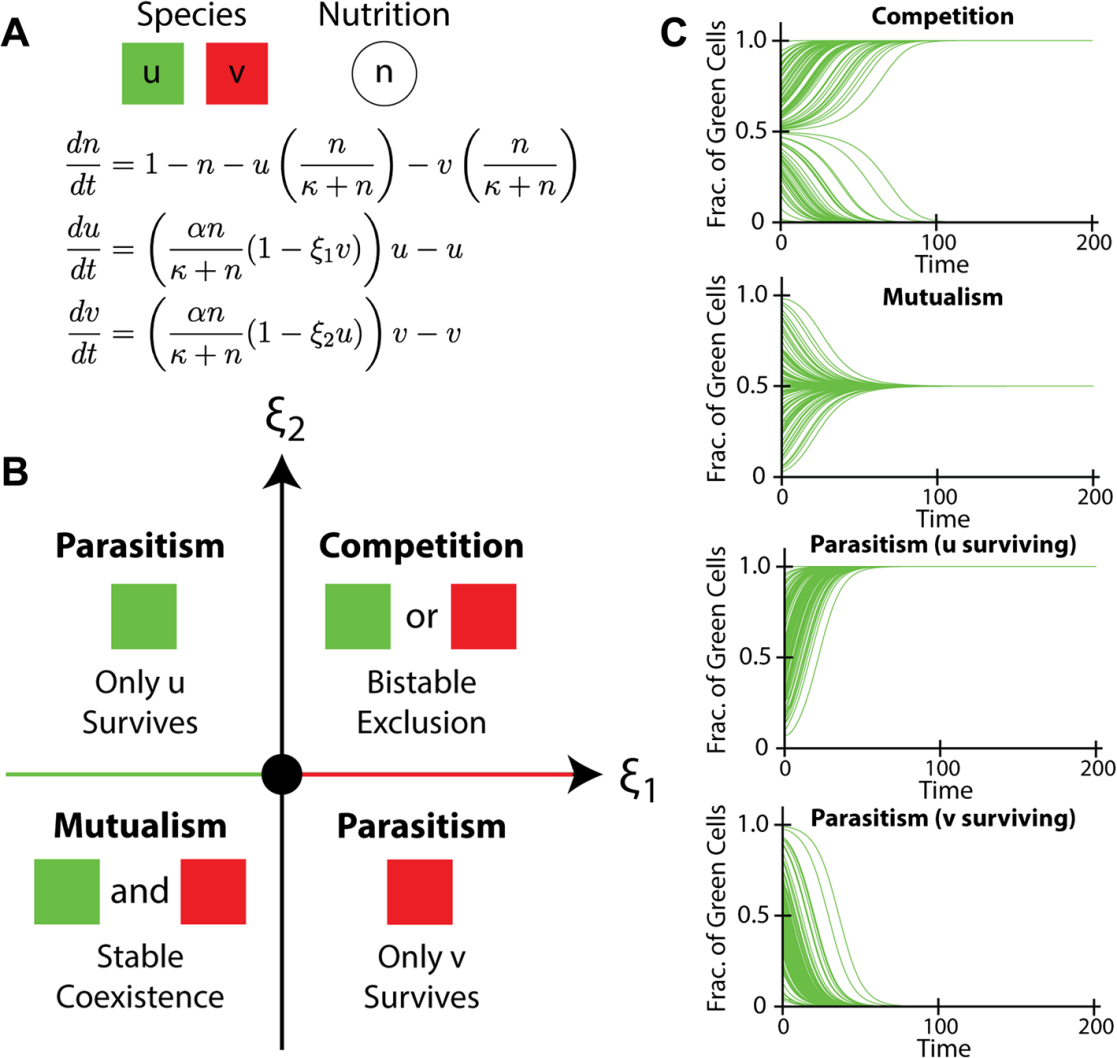
Analysis of a two-species community in the well-mixed case. **a** An ordinary differential equation model describing the growth dynamics of two species, including nutrient consumption and interspecies interactions, and a shared nutrition source. **b** Phase diagram for the steady states of the model. Qualitatively distinct outcomes may arise from communities with different interactions. Competition results in bistable exclusion, mutualism leads to stable co-existence, and parasitism leads to the survival of the species with growth advantage. The origin, green colored axis and red colored axis correspond to the cases of control (neutralism), commensalism, and amensalism respectively. **c** Simulations of the population dynamics for the communities that are competing, mutualistic, and parasitic

To gain analytical insights into our findings regarding community structures and social interactions, we considered a simplified version of bacterial colony expansion–a two-species, well-mixed community. As shown in Fig. 6a, a mathematical model was constructed using ordinary differential equations (ODEs) to describe the population dynamics of the system. In the ODEs, *n* represents a shared nutrition source, with *u* and *v* as two interacting cellular species. As in the full spatial model, the two species interact through asocial (consumption of a shared nutrient, $\frac {\alpha n}{\kappa +n}$) and social (production of toxins or public goods, 1−*ξ*_1_*v* and 1−*ξ*_2_*u*) interactions. The social interactions are quantified by the parameters *ξ*_1_ and *ξ*_2_. For the well-mixed case, the production of diffusible chemicals is assumed to be at steady-state so that the interaction takes place through the cell density. Thus, the simplified ODEs mimic the full spatial model and allow us to enhance our interpretation of the results.

From the equations (Fig. 6a), we derived a phase diagram (Fig. 6b, Additional file 1: Figure S12, Additional file 1: Section 2) that describes the outcomes of the well-mixed community. We found that, for the simplified model, competition causes species exclusion although either of the two may win, mutualism results in stable co-existence, and parasitism leads to survival of only the parasitic species. To confirm the results, we also performed multiple runs of the ODE model with varying initial conditions for each of the cases (Fig. 6c and Additional file 1: Figure S12), showing good consistency with the phase diagram.

Although the simplified model neglects spatial information, it does provide a set of valuable insights into our findings. In this model, parasitism, commensalism and amensalism all lead to the exclusive survival of the species with higher fitness in a community, qualitatively consistent with the findings from the computational framework that the structures of the communities with asymmetrical interactions have a biased abundance towards the species with a growth advantage. In contrast, competition, mutualism, and neutralism in the simple model result in the extinction of either species with an even chance, perfect co-existence of the two species, and an initial condition-dependent random abundance respectively, all of which have an equal relative species abundance upon ensemble average (Additional file 1: Table S9). These results are also in agreements with the equal relative abundance observed in the spatial structures of the communities with symmetrical interactions.

Additionally, given random initial conditions, competing communities always produce a steady species ratio of either 1:0 or 0:1; mutualistic communities always result in an equal 1:1 ratio, while neutralistic communities lead to a random species ratio between 1:0 and 0:1. Therefore, although the ensemble averages of the relative abundances for the three symmetrical cases (competition, mutualism, and neutralism) are the same, their variances shall be distinct with that of mutualism being minimal and that of competition being maximal (Additional file 1: Table S9). This result is again qualitatively consistent with the order of the variances of the relative abundances for competing, mutualisitic and neutral communities in the spatial setting.

In addition to relative abundance, the simplified model can also be applied to understand the number of surviving lineages for the spatially expanding colonies. In the spatial case, the numbers of surviving lineages for the mutualisitic, neutral, and competing communities follow an order from high to low. This is because the stable co-existence for mutualism contributes to an enhancement in total lineages at the expanding front. In contrast, the exclusive nature of the competitive interaction drives surrounding species to extinction, resulting in decreased diversity at the population edge.

## Conclusions

In this work, we performed a systematic survey on the impacts of social interactions on the spatial structure of bacterial communities. We developed and utilized a hybrid community modeling framework that combines discrete element techniques with reaction-diffusion equations–the former for cellular force calculation and the latter for social interaction computation. We found that cellular social interactions have a profound impact on bacterial communities, with different interactions leading to qualitatively distinct characteristics for colony structures. Specifically, deleterious interactions (amensalism and competition) can cause an increased variance in relative abundance relative to the mean, a drastic decrease in the number of surviving lineages, and a rough expanding front; by contrast, beneficial interactions (commensalism and mutualism) contribute to a reduced variance in abundance relative to the mean, an enhancement in lineage number, and a smooth expanding front. In addition, the communities with asymmetric interactions have a differential mean relative abundance while those with symmetric interactions differ in the variance of their relative abundance. Moreover, for the communities with symmetric interactions, mutualism promotes spatial homogeneity and population robustness while competition increases spatial segregation and population fluctuations.

Due to the focus of this work on the interaction-structure relationship as well as the computationally intensive nature of the simulation framework, certain features of natural populations were simplified. For instance, toxin and public good production were assumed to be constitutive, all cells were constrained to move within two-dimensional space, and the number of cells within a growing population was limited to 30,000. It will thus be valuable to relax these constraints to study the interaction-structure relationship in more complex settings in the future. For example, social interactions, implemented through the production of toxins or public goods, are often subject to density-dependent mechanisms such as quorum sensing. It will be interesting to examine how the density dependence of cellular behaviors shapes our conclusions regarding community structures. Additionally, our framework allows the analysis of cellular density over time as shown in Additional file 1: Figures S13–S24, which provides the capacity to systematically examine the role of density-dependent cellular behaviors in impacting community structures.

In summary, this work provides a quantitative and statistical picture of the relationship between bacterial social interactions and spatiotemporal community structures. Such a picture will allow a comprehensive understanding of the roles of pairwise social interaction, which sets a basis for understanding more complex microbial communities such as biofilms and the microbiome. Therefore, this study advances our fundamental understanding of microbial sociobiology and community structure. In addition, from an engineering viewpoint, our systematic study benefits the design and construction of synthetic microbial consortia. For instance, the increased robustness of population structure in a mutualistic community compared to that of competitive and neutral communities suggests that researchers need to design cooperative ecosystems for robust performance of desired cellular functionality. Such knowledge will be instrumental for engineering artificial microbial consortia towards various applications.

## Methods

### Cell growth and division

Cellular growth rate is determined by the local concentrations of related chemicals including nutrients, toxins, and public goods. The Monod equation [39] is used to model the dependence of cellular growth on nutrients, while for toxins and public goods, a linear relationship is assumed. Cell length expansion is based on growth rate and cell area until division [40] using the following equation for a given cell *i*:

(1) $$ \frac{dl_{i}}{ dt}= gA_{i}\frac{n}{\kappa+n}\left(1-\xi T \right)  $$

where *l*_*i*_ is the cell length, *g* is the growth rate, *A*_*i*_ is the cell area, *n* is nutrient concentration, *κ* is nutrient sensitivity, *T* is toxin or public good concentration, and *ξ* is the interaction strength. Any possible negative values for the derivative of *l*_*i*_ with respect to time were set to zero. The dependence of all cells in the simulation on the same nutrition source (*n*) leads to a slower growth rate in the high density, nutrient depleted core of the expanding population than on the edge. The total length at division follows a truncated normal distribution (mean of 4.0 *μ*m and standard deviation of 0.3 *μ*m), with a random division length assigned to each cell upon creation. Values outside of [3.1,4.9] were set to the respective extreme values.

### Mechanical cell interactions

Intercellular mechanical forces are calculated using a soft particle technique [41] with the following equation [42]:

(2) $$ F_{ij} = \left\{ \begin{array}{lr} Ed^{\frac{1}{2}}h_{ij}^{\frac{3}{2}} & : h_{ij} > 0\\ 0 & : h_{ij} \le 0 \end{array} \right.  $$

where *h*_*ij*_ is the overlap between spheres of diameter *d* placed at the closest points between cells *i* and *j*; *E* is an elasticity constant. The spheres have a radius identical to that of the corresponding cell, making sphere overlap equivalent to cell overlap. The resulting force is normal to the plane of contact, and forces are applied on the axis of each respective cell, resulting in both translational and rotational motion following Newton’s Laws. All motion for the simulations was constrained to two-dimensional space (see Additional file 1: Section 1.1).

The parameter values used in the simulation to model cells expanding on a solid substrate (see Additional file 1: Section 1.1 and [33]) allow us to neglect the inertial terms in the equations of motion. Thus, the motion for a given cell (*i*) is determined by the following equations:

(3) $${} \frac{d\vec{q}_{i}}{d\tau} = \sum\limits_{contacts}\left(\frac{Et_{c}}{\beta\rho }\right)\left(\frac{(1- |\vec{q}_{ci}-\vec{q}_{cj}|)^{\frac{3}{2}}}{L_{i}}\right)\hat{q}_{ij}  $$

(4) $${} \frac{d\phi_{i}}{d\tau} = \sum\limits_{contacts}\left(\frac{Et_{c}}{\beta\rho }\right)\left(\frac{12(1- |\vec{q}_{ci}-\vec{q}_{cj}|)^{\frac{3}{2}}}{{L_{i}^{3}}}\right)\hat{q}_{ij}\times (\vec{q}_{ci}-\vec{q}_{i})  $$

where $\vec {q}_{i}$ is the center of mass position of the cell in two-dimensional space, *ϕ*_*i*_ is the orientation of the cell, $\vec {q}_{\textit {ci}}$ and $\vec {q}_{\textit {cj}}$ are the closest points of contact between cells *i* and *j*, $\hat {q}_{\textit {ij}}$ is a unit vector in the direction of ($\vec {q}_{\textit {ci}} - \vec {q}_{\textit {cj}})$, *L*_*i*_ is the length of the cell, and (*Et*_*c*_/ *βρ*) is a dimensionless parameter depending on the elasticity (*E*), chosen timescale (*t*_*c*_), viscous drag (*β*), and mass density per unit length for the cell (*ρ*). The sum over contacts consists of cells that have a nonzero contact force (i.e. *h*_*ij*_>0). For all of the spatial variables, the length scale is chosen to be 1 *μ*m. The time scale is chosen to be 30 min. See the Additional file 1: (Table S1 and Section 1.1) for a full list of all parameter values used and a derivation of the equations of motion.

### Chemical diffusion

The spatiotemporal distribution of chemicals is subject to diffusion as well as system-specific reactions with other chemicals and cells at a given spatial grid point. In general, the time evolution of a chemical *c* is determined by:

(5) $$ \frac{dc}{dt} = D_{c}\nabla^{2}c + \alpha_{c}f(\rho_{c},c) - \beta_{c} c  $$

where *D*_*c*_ is the diffusion constant, *α*_*c*_ and *f*(*ρ*_*c*_,*c*) determine production (or consumption) by cells, *ρ*_*c*_ is the density of cells that interact with the chemical, and *β*_*c*_ determines degradation. The parameters are chosen to give realistic values for the active layer of growing cells during expansion and a well-defined spatial scale for interactions. (see Additional file 1: Section 1.2).

In the framework, nutrient is a diffusible chemical species that is shared by all of the cells. A constant boundary condition is assumed for the nutrient to represent an external source. The time evolution equation for nutrient is given by:

(6) $$ \frac{dn}{dt} = D_{n}\nabla^{2}n - \alpha_{n}\frac{\rho n}{\kappa+n}  $$

where *n* is nutrient concentration, *D*_*n*_ is the diffusion constant, *α*_*n*_ is the cell consumption rate, *κ* is the nutrient sensitivity, and *ρ* is the total density of all species at a grid point in space, defined as the area of all cells within a grid divided by the area of the grid. The value for nutrition is scaled relative to the constant nutrient boundary condition. See Additional file 1: Section 1.2 for a full list of parameter values used.

For other chemicals, which are produced by cells, a reactive boundary condition is assumed to account for flow out of the system and production is assumed to be constitutive. The boundary conditions, however, shall have negligible effects on the time evolution of the system for a sufficiently large spatial area. The time evolution equations for produced chemicals are given by:

(7) $$ \frac{dc}{dt} = D_{c}\nabla^{2}c + \rho_{c} - \beta_{c} c  $$

where *c* is the chemical concentration under consideration, *D*_*c*_ is the diffusion constant, *ρ*_*c*_ is the density of producing cells at a grid point in space, and *β*_*c*_ determines degradation. The concentration *c* is scaled so that the production term does not have an additional parameter. Notice that *D*_*c*_ and *β*_*c*_ set a length scale for interactions mediated by the diffusible chemical. See Additional file 1: Section 1.2 for a full list of parameter values used.

### Simulation protocols

A 60 by 60 *μm*^2^ square was used for initial seeding. For random initial conditions, cellular orientation, length, number, and species type at each grid were randomly chosen but the overall species abundance and cell number were preserved to follow specific simulation requirements. For well-mixed initial conditions, species were placed in an alternating pattern in grids with the overall species abundance and cell number following defined requirements. A total of 256, 64, and 16 initial cells were used to represent the case of high, medium, and low initial cell densities. All of the initial conditions tested were generated using Mathematica. All of the simulations exit when the overall cell number reaches 30,000 cells. Values for all parameters in the simulations can be found in Additional file 1: Section 1.

### Quantitative metrics

Colony roughness was used as a measure for the morphology of a bacterial population. It was determined by first locating the edge of the colony–Each spatial grid point containing at least one cell was considered occupied and the occupied grids with empty nearest neighbors were classified as the edge. Subsequently, the center of the colony was calculated by averaging over the positions for all cells, weighted by the two-dimensional area for each cell. Afterwards, the mean and standard deviation of the edge grid points from the colony center were computed. Here, the standard deviation serves as the quantitative metric of the colony roughness, whose scale is the size of one grid point (side length of 5 *μ*m). The mean gives an estimate of the colony radius, which can also be estimated by calculating the moment of inertia of the colony and solving for the radius assuming a disc with constant density (as in Fig. 4).

Surviving lineages are defined as the lineages that remain active. We thus first identified active cells in a community–In our analysis, we considered a cell to be currently active if it has a nutrient availability that allows its growth rate to be within two exponential decays of the maximum. Meanwhile, as the lineage information of every cell is retained during a single simulation, we leveraged this information to identify the ancestors of the active cells. The number of ancestors counted is the number the surviving lineages.

Similar to surviving lineages, we determined the colony sectors by considering the layer of actively growing cells. First, the image of the actively growing cells in the final simulation snapshot was imported in Mathematica. The remaining pixels were directly set as black in Mathematica. Then, a convolution was performed with a constant kernel which averages nearest neighbor pixels. Afterwards, each pixel was classified as either green, red, or background based on the dominant color (red, green, or completely black). Finally, spatially connected components and the number of pixels for each component for the figure were obtained. The number of sectors was determined by counting the number of pixel clusters representing groups of more than 100 cells. The sector analysis was performed for the final population states at 30,000 cells.

In addition to the metrics used to quantify the spatial structures of the expanding colonies (i.e. relative abundance, morphology, surviving lineages, and colony sectors), we have also considered the time evolution of the cell density. As shown in Additional file 1: Figures S13–S21, the total cell density, defined as the number of cells per unit area in two-dimensional space, follows a somewhat standard trajectory in time. After an initial transient period, the cells form a dense quasi-circular structure with a well-defined edge. Within the expanding colony, the density is approximately constant, representing a well-packed spatial limit, while the density decreases rapidly to zero at the colony edge. Also shown in Additional file 1: Figures S13–S21, the density for each individual cellular species shows the formation of distinct spatial structures according to interaction type. Given the two-dimensional nature of the simulations, the cell density at a given time can be inferred from images showing each cell colored according to type (Additional file 1: Figures S22–S24). The metrics analyzed and discussed throughout the text (e.g. Figure 4) are used to give a thorough characterization of the spatial structures produced by each interaction type over time.

### Implementation

Simulations in the work were implemented using C++, with additional testing and prototyping performed in Mathematica. OpenMP was used for parallelization of the code, with parallel force calculations utilized for the simulation of large systems. The default container used to hold data was an instance of the standard vector class. Each cell in the simulation constituted an instance of a particle class including data members position, velocity, force, length, cell type, and growth rate.

### Visualization and analysis

All data concerning cells, nutrients, and toxins were written to files for later analysis and visualization. Individual frames were rendered in VMD [43], a program commonly used for visualizing molecular dynamics. Colony data analysis was performed in Mathematica, with customized functions developed to determine species abundance, colony morphology, cell lineages, and segregated sectors. Details of the methods are available in the Supporting Information.

## Additional file

Additional file 1
**Supporting Information.** The file contains two text sections detailing the equations and parameters used for simulations and ODE analysis. Figures S1–S24 and Tables S1–S9 are included with further simulation data.
