# Supplementary material for: Bacterial social interactions drive the emergence of differential spatial colony structures
Source: BMC Syst Biol. 2015 Sep 16;9:59. doi: 10.1186/s12918-015-0188-5 (PMC4573487; doi:10.1186/s12918-015-0188-5)
Supplement: Additional file 1 — Supporting Information. The file contains two text sections detailing the equations and parameters used for simulations and ODE analysis. Figures S1–S24 and Tables S1–S9 are included with further simulation data. [file 12918_2015_188_MOESM1_ESM.pdf]

Supporting information for:
Bacterial social interactions drive the emergence of differential spatial colony structures

Andrew E. Blanchard[†] and Ting Lu[*,‡,†,¶]

Department of Physics, University of Illinois at Urbana-Champaign, IL 61801, U.S.A., Department of Bioengineering, University of Illinois at Urbana-Champaign, IL 61801, U.S.A., and Institute for Genomic Biology, University of Illinois at Urbana-Champaign, IL 61801, U.S.A.

E-mail: luting@illinois.edu

[*]To whom correspondence should be addressed
[†]Department of Physics, University of Illinois at Urbana-Champaign, IL 61801, U.S.A.
[‡]Department of Bioengineering, University of Illinois at Urbana-Champaign, IL 61801, U.S.A.
[¶]Institute for Genomic Biology, University of Illinois at Urbana-Champaign, IL 61801, U.S.A.

1 Computational Framework

1.1 Mechanical Forces

Time Evolution Equations: In a two-dimensional space, a bacterial cell (i) can be modeled as a rigid rod with center of mass coordinates ($\vec{r}_i$), center of mass velocities ($\vec{v}_i$), orientation with respect to some specified axis (ϕ_i), density (ρ_i), and length (l_i). Here we assume that the intracellular density is constant for all cells ($\rho_i = \rho$) so that the mass of a given cell can be calculated using:

$$m_i = \rho l_i \tag{1}$$

With this assumption, we can write down the fundamental equations that govern cellular motion, namely Newton's laws, for the force and torque of a rigid body moving in a viscous substance:

$$\rho l_i \frac{d^2\vec{r}_i}{dt^2} = \vec{F}_i(\{\vec{r}\}, \{\vec{v}\}) - \beta \rho l_i \frac{d\vec{r}_i}{dt} \tag{2}$$

$$\frac{\rho l_i^3}{12} \frac{d^2\phi_i}{dt^2} = \tau_i(\{\vec{r}\}, \{\vec{v}\}) - \frac{\beta \rho l_i^3}{12} \frac{d\phi_i}{dt} \tag{3}$$

where the brackets ($\{\}$) denote a function of all coordinates and velocities and β denotes the drag from the surrounding viscous medium. The torque is calculated from the cross product of the the lever arm and the corresponding force.

The unique aspects of simulating a bacterial population lie in the facts that cells grow and divide, that the particle number of the system is not conserved, and that l_i is a function of time. In order to successfully model the dynamics of such a system, the division time for a given bacterial cell is chosen as the typical time scale of the system and the diameter of the cell is chosen as the basic spatial length scale. Here we introduce two corresponding new

variables:

$$\vec{r}_i = r_c \vec{q}_i$$
$$t = t_c \tau \tag{4}$$

where r_c and t_c have dimensions of length and time respectively. With this change of variables, length in the system can be written as

$$l_i = r_c L_i \tag{5}$$

Our equations of motion can thus been written as

$$\frac{\rho r_c^2 L_i}{t_c^2} \frac{d^2 \vec{q}_i}{d\tau^2} = \vec{F}_i - \frac{\beta \rho r_c^2 L_i}{t_c} \frac{d\vec{q}_i}{d\tau} \tag{6}$$
$$\frac{\rho r_c^3 L_i^3}{12 t_c^2} \frac{d^2 \phi_i}{d\tau^2} = \tau_i - \frac{\beta \rho r_c^3 L_i^3}{12 t_c} \frac{d\phi_i}{d\tau} \tag{7}$$

where the forces and torques are also functions of dimensionless variables. Such nondimensionalization allows to arrange the terms in a way that emphasizes the contribution of the inertia term to the overall motion as

$$\frac{1}{\beta t_c} \frac{d^2 \vec{q}_i}{d\tau^2} = \frac{t_c}{\beta \rho r_c^2 L_i} F_i - \frac{d\vec{q}_i}{d\tau} \tag{8}$$
$$\frac{1}{\beta t_c} \frac{d^2 \phi_i}{d\tau^2} = \frac{12 t_c}{\beta \rho r_c^3 L_i^3} \tau_i - \frac{d\phi_i}{d\tau} \tag{9}$$

To implement simulation, we need to specify the force and torque; however, we notice there are now two clear timescales for the system, namely $1/\beta$ which governs that amount of time for a given particle to change velocity and another timescale associated with the force which governs the typical time for an interaction. The detailed forms are presented below.

Hertzian Forces: The following equation for the magnitude of contact force was used

to describe the expansion of a bacterial colony on a solid substrate (S1) .

$$F_{ij} = \begin{cases} Ed^{\frac{1}{2}} h_{ij}^{\frac{3}{2}} & : h_{ij} > 0 \\ 0 & : h_{ij} \leq 0 \end{cases}$$

where E is a constant describing the rigidity of the rod, d is the interaction diameter of the rod, and $h_{ij} = d - |\vec{r}_{ci} - \vec{r}_{cj}|$. Here, $\vec{r}_{ci}$ and $\vec{r}_{cj}$ denote the closest points between the two rods. In addition, the direction of the force is in the same direction as $\vec{r}_{ci} - \vec{r}_{cj}$. Using this force form, the equations of motion for rods in contact can be expressed as:

$$\begin{aligned} \frac{1}{\beta t_c} \frac{d^2 \vec{q}_i}{d\tau^2} &= \sum_{contacts} \frac{E d^{\frac{1}{2}} t_c (d - r_c |\vec{q}_{ci} - \vec{q}_{cj}|)^{\frac{3}{2}}}{\beta \rho r_c^2 L_i} \hat{q}_{cij} - \frac{d\vec{q}_i}{d\tau} \\ \frac{1}{\beta t_c} \frac{d^2 \vec{q}_i}{d\tau^2} &= \sum_{contacts} \frac{E t_c (1 - |\vec{q}_{ci} - \vec{q}_{cj}|)^{\frac{3}{2}}}{\beta \rho L_i} \hat{q}_{cij} - \frac{d\vec{q}_i}{d\tau} \\ \frac{1}{\beta t_c} \frac{d^2 \vec{q}_i}{d\tau^2} &= \sum_{contacts} \left(\frac{E t_c}{\beta \rho} \right) \left(\frac{(1 - |\vec{q}_{ci} - \vec{q}_{cj}|)^{\frac{3}{2}}}{L_i} \right) \hat{q}_{cij} - \frac{d\vec{q}_i}{d\tau} \end{aligned} \tag{10}$$

where the sum over contacts includes cells that have a nonzero contact force (i.e. $h_{ij} > 0$). Similarly, the equations for torque can be written as:

$$\frac{1}{\beta t_c} \frac{d^2 \phi_i}{d\tau^2} = \sum_{contacts} \left(\frac{E t_c}{\beta \rho} \right) \left(\frac{12(1 - |\vec{q}_{ci} - \vec{q}_{cj}|)^{\frac{3}{2}}}{L_i^3} \right) \hat{q}_{cij} \times (\vec{q}_{ci} - \vec{q}_i) - \frac{d\phi_i}{d\tau} \tag{11}$$

From the above equations, we can explicitly see the two timescales in the problem, namely:

$$\begin{aligned} t_1 &= \frac{\beta \rho}{E} \\ t_2 &= \frac{1}{\beta} \end{aligned} \tag{12}$$

Alternatively, we can use the combined parameter $\zeta = \beta\rho$ instead. For bacteria (*E. coli*)

growing on a solid substrate, the estimated values are (S1)

$$\begin{aligned}\zeta &= 200\ \text{Pa}\cdot\text{hr}\\ E &= 4\times 10^{6}\ \text{Pa}\end{aligned}\tag{13}$$

To estimate t_1 and t_2, we also need the density of a bacterial cell which can be approximated as

$$\begin{aligned}\rho &\approx \frac{10^{-15}\text{kg}}{3\times 10^{-6}\text{m}}\\ &\approx 3\times 10^{-10}\ \text{kg/m}\end{aligned}\tag{14}$$

From the above estimates, we can obtain the two time scales as

$$\begin{aligned}t_1 &\approx 5\times 10^{-5}\text{hr}\\ t_2 &\approx 1.5\times 10^{-12}\text{hr}\end{aligned}\tag{15}$$

from which we find that the timescale for the velocity to change is much shorter than the timescale for cellular interaction. Therefore, for a practical simulation, it is thus appropriate to neglect the inertial term and choose a longer physical time step, which results in the first order equations for force and torque below:

$$\begin{aligned}\frac{d\vec{q}_i}{d\tau} &= \sum_{contacts}\left(\frac{Et_c}{\beta\rho}\right)\left(\frac{(1-|\vec{q}_{ci}-\vec{q}_{cj}|)^{\frac{3}{2}}}{L_i}\right)\hat{q}_{ij}\\ \frac{d\phi_i}{d\tau} &= \sum_{contacts}\left(\frac{Et_c}{\beta\rho}\right)\left(\frac{12(1-|\vec{q}_{ci}-\vec{q}_{cj}|)^{\frac{3}{2}}}{L_i^3}\right)\hat{q}_{ij}\times(\vec{q}_{ci}-\vec{q}_i)\end{aligned}\tag{16}$$

Notably, the timescale for interactions (t_1) also provides a point of reference for choosing the integration timestep during simulations because it tells the order of magnitude for time that two rods will contact. Thus, to ensure successful simulation of cellular collisions, we shall

choose a timestep that is smaller than t_1. A reasonable choice would be:

$$\begin{aligned}\Delta\tau &= \frac{t_1}{10t_c} \\ &= 10^{-5}\end{aligned} \tag{17}$$

where we used 0.5 hr (typical division time for *E. coli* in exponential growth) for t_c. Notice that this corresponds to the interval in real time:

$$\Delta t = 5 \times 10^{-6}\text{hr} \tag{18}$$

It is important to note that this separation of timescales for t_1 and t_2 is a direct consequence of slow movement of cells on a solid substrate. For liquid environments, the timescales may be of similar magnitude during which eliminating the acceleration term in the equations of motion will not be appropriate.

Numerical Integration: The Euler method was used to integrate the time evolution of the force and torque equations. Due to the differences in growth rates of the cells with different interactions, we found it helpful to choose a variable timestep instead of choosing a minimal fixed time interval. The variable timestep was determined by ensuring that the maximal movement of any cell was a small fraction of the cell diameter. This strategy enabled substantially faster simulations for the case of populations with slow overall growth rates due to deleterious interactions (i.e. competition). The force calculations were implemented utilizing the following procedures: (1) cells were classified to a spatial grid; (2) cells in nearest neighbor grids were tested for possible overlaps by finding the closest points for each respective pair of cells; (3) cells that overlapped were assembled into a list; (4) forces and torques at the closest contact points were calculated in parallel and output to a force list; (5) forces and torques were combined for each cell. Some sample code from (S2) was used to aid in development.

Table S1: Summary of Mechanical Force Equations

Description	Equation
Forces	$\frac{d\vec{q}_i}{d\tau} = \sum_{contacts} \left(\frac{Et_c}{\beta\rho}\right) \left(\frac{(1 - \lvert\vec{q}_{ci} - \vec{q}_{cj}\rvert)^{\frac{3}{2}}}{L_i}\right) \hat{q}_{ij}$
Torques	$\frac{d\phi_i}{d\tau} = \sum_{contacts} \left(\frac{Et_c}{\beta\rho}\right) \left(\frac{12(1 - \lvert\vec{q}_{ci} - \vec{q}_{cj}\rvert)^{\frac{3}{2}}}{L_i^3}\right) \hat{q}_{ij} \times (\vec{q}_{ci} - \vec{q}_i)$

Description	Parameter	Value
Length Scale	r_c	1 μm
Time Scale	t_c	30 min
Cell diameter	d	1 μm
Elasticity constant	E	4×10^6 Pa
Drag constant	$\zeta = \beta\rho$	200 Pa $\cdot$ hr

1.2 Diffusible Chemicals

Time Evolution Equations: Every chemical (c) in the system is governed by a corresponding reaction-diffusion equation as

$$\begin{aligned} \frac{dc}{dt} &= D_c \nabla^2 c + \alpha_c f(\rho_c, c) - \beta_c c \quad \in \quad \Omega \\ c &= c_0 \quad \in \quad \partial\Omega \end{aligned} \tag{19}$$

where D_c is the diffusion constant, α_c and $f(\rho_c, c)$ determine production (or consumption) by cells, ρ_c is the density of cells that interact with the chemical, and β_c determines degradation. Ω is a two-dimensional rectangular region and $\partial\Omega$ is the boundary; c_0 is the concentration on the boundary. For the shared nutrition source (n), we assume that there is no auto-degradation (i.e. $\beta_n = 0$). We also re-scale the nutrition concentration so that its value on the boundary equals 1. For the chemicals that mediate cellular interactions, we impose a reactive boundary ($c_0 = 0$). Similarly, we scale the concentration so that $\alpha_c = 1$. Moreover, in all cases, the two dimensional region simulated is much larger than the colony size, so that

the reactive boundary condition for the diffusible chemicals should have negligible effects.

Nutrition: Based on the Monod function (S3), the exact equation for nutrition is given as

$$\frac{dn}{dt} = D_n \nabla^2 n - \alpha_n \frac{\rho n}{\kappa + n} \tag{20}$$

where n is nutrition, ρ is the total density of all species at a grid point in space, defined as the area of all cells within a grid divided by the area of the grid. Parameters were chosen to approximately reproduce the experimentally determined width of actively growing cells of bacterial colonies (30-40μm) (S4), which are in agreement with previous simulations (S1). The non-dimensional values are $D_n = 250$, $\alpha_n = 1$, and $\kappa = 0.333$. The boundary of the simulated area serves as a constant source of nutrient.

Chemicals for Interactions: For chemicals other than nutrition, its production by a given species is assumed constant. Therefore, the corresponding dimensionless reaction-diffusion equation has a form of

$$\frac{dc}{dt} = D_c \nabla^2 c + \rho_c - \beta_c c \tag{21}$$

where c is the chemical, ρ_c is the density of the producer species at a grid point in space. In this work, the diffusion (D_c) and degradation (β_c) parameters ($D_c = 1000$ and $\beta_c = 10$) were chosen so that cellular interaction range is on the order of few spatial grid points. Notably, for a well occupied spatial grid in the absence of diffusion, the steady state value for a given chemical follows

$$\begin{aligned} 0 &= \rho_c - \beta_c c \\ 0 &= 1 - \beta_c c \\ c &= \frac{1}{\beta_c} \end{aligned} \tag{22}$$

which provides an estimate for the level of chemical within the densely packed region of the expanding colony.

Numerical Integration: The Euler method was employed for simulating all of the reaction-diffusion equations. A discretized version of the diffusion operator from a Taylor expansion to second order in grid size was used. A grid size of 5 was used throughout simulations, which is comparable to the max length for a single cell. The simulated region was a square with a side length of 1000, which is much larger than the typical final radius of the colonies (≈ 200)

Table S2: Summary of Diffusion Equations. The value for nutrient sensitivity is taken with respect to the maximal concentration at the simulation boundary. Degradation, consumption, and diffusion parameters were chosen to give a desired length scale for active cell growth and interactions.

Description	Equation
Nutrients	$\frac{dn}{dt} = D_n \nabla^2 n - \alpha_n \frac{\rho n}{\kappa + n}$
Interactions	$\frac{dc}{dt} = D_c \nabla^2 c + \rho_c - \beta_c c$

Description	Parameter	Value
Nutrient diffusion	D_n	500 μm^2/hr
Nutrient consumption	α_n	2 hr^{-1}
Nutrient sensitivity	κ	0.333
Toxin/Public good diffusion	D_c	2000 μm^2/hr
Toxin/Public degradation	β_c	20 hr^{-1}

1.3 Cell Growth and Division

Cellular growth follows the following equation:

$$\frac{dl_i}{dt} = g A_i \frac{n}{\kappa + n} (1 - \xi T) \tag{23}$$

where l_i is the length of the cell (i), A_i is the cell area, g is the maximal growth rate, κ is the parameter associated with nutrient consumption (0.333 as in section 1.2), ξ is an interaction parameter, and T is the concentration of diffusible chemical produced by the other species. This specific form for elongation is similar to that found in (S1).

As discussed previously, we expect the maximal concentration of the diffusible chemicals to be approximately $1/\beta_c$ (or 1/10 in the simulations) for a well-packed spatial grid of producing cells. We thus chose the interaction parameter ξ so that the exposure of the cell to the maximal concentration resulted in either cessation of cell growth (toxin) or doubling of cell growth (public good) ($\xi = +10$ or -10 respectively). To ensure that the cells in the population never decrease in length, the growth rate was truncated to zero for any possible negative rates that arose.

The growth constant g was determined by considering the case of nutrient saturation ($n = 1$) at which the doubling time is approximately one time unit in the absence of other diffusible chemicals, i.e.,

$$\begin{aligned} \frac{dl_i}{dt} &= gA_i \frac{1}{\kappa+1} \\ &= \frac{g}{\kappa+1}\left(\frac{\pi}{4} + l_i\right) \\ l_i &= \left(l_0 + \frac{\pi}{4}\right) e^{\frac{g}{\kappa+1}t} - \frac{\pi}{4} \end{aligned} \tag{24}$$

Calculating the time for a cell to grow from a length of 1.5 to 4.0 for division gives:

$$\begin{aligned} e^{\frac{g}{\kappa+1}t} &= \frac{4+\frac{\pi}{4}}{1.5+\frac{\pi}{4}} \\ t &= \frac{\kappa+1}{g}\ln\left(\frac{4+\frac{\pi}{4}}{1.5+\frac{\pi}{4}}\right) \end{aligned} \tag{25}$$

Finally, by setting the above time as 1 and plugging in the parameter value of κ, we obtained

$$
\begin{aligned}
g &= (1.333)(0.739) \\
g &\approx 1
\end{aligned}
\tag{26}
$$

For cellular division, we assumed that each cell has a length that increases over time until it reaches a specified division length. The division length for a cell was generated randomly from a Gaussian distribution with a mean of 4 and a standard deviation of 0.3. Any values for division length outside of $[3.1, 4.9]$ were truncated to the extreme values. Notably, the total of cell length plus the mechanical interaction range is conserved during division so that newly divided cells did not overlap. Each daughter cell received a fraction of the parent length between 0.4 and 0.6 (adding up to one in total) randomly.

2 ODE Analysis

To interpret the results obtained from the simulation of our community modeling framework, we considered a simplified case of the system–a well-mixed model. As shown below, the model consists of three variables, including nutrition (n) and the populations of two cellular species (u and v).

$$
\begin{aligned}
\frac{dn}{dt} &= (n_0 - n)D - \frac{u}{\gamma}\left(\frac{mn}{\kappa + n}\right) - \frac{v}{\gamma}\left(\frac{mn}{\kappa + n}\right) \\
\frac{du}{dt} &= \left(\frac{mn}{\kappa + n}(1 - \xi_1 v)\right) u - Du \\
\frac{dv}{dt} &= \left(\frac{mn}{\kappa + n}(1 - \xi_2 u)\right) v - Dv
\end{aligned}
\tag{27}
$$

The parameters are nutrient supply (n_0), degradation or flow rate (D), nutrient consumption (m), consumption to growth ratio (γ), nutrient sensititivey (κ), and interspecies interaction strength (ξ_1 and ξ_2). Notice that the first term on the right hand side of the equations for both u and v consists of nutrient consumption and interaction terms; the second term results form death or flow out of the system. As we are primarily interested in the system's steady states and their stabilities, we performed the change of variables using

$$
\begin{aligned}
t &= \frac{\bar{t}}{D} \\
n &= n_0 \overline{n} \\
u &= \frac{n_0 \gamma D}{m} \overline{u} \\
v &= \frac{n_0 \gamma D}{m} \overline{v}
\end{aligned}
\tag{28}
$$

which results in the following equations after dropping the bars and rescaling the parameters:

$$\begin{aligned}\frac{dn}{dt} &= 1 - n - u\left(\frac{n}{\kappa + n}\right) - v\left(\frac{n}{\kappa + n}\right) \\ \frac{du}{dt} &= \left(\frac{\alpha n}{\kappa + n}(1 - \xi_1 v)\right) u - u \\ \frac{dv}{dt} &= \left(\frac{\alpha n}{\kappa + n}(1 - \xi_2 u)\right) v - v \end{aligned} \tag{29}$$

The steady states of the system can then be derived by setting the above equations to zero. When considering the solutions to the equations, there are three possible types: u and v both are zeros; one of them is zero; and neither of them is zero. For the case where both are zero, the steady state solution is:

$$\begin{aligned} n &= 1 \\ u &= 0 \\ v &= 0 \end{aligned} \tag{30}$$

For the case where v is extinct and u is nonzero, the steady state solution is:

$$\begin{aligned} n &= \frac{\kappa}{\alpha - 1} \\ u &= \alpha\left(1 - \frac{\kappa}{\alpha - 1}\right) \\ v &= 0 \end{aligned} \tag{31}$$

Similarly, the steady state solution for the case of u is extinct and v is nonzero can also be expressed. Furthermore, for the case where both are nonzero, the detail expression can also be derived (It is not listed here as the expression is quite cumbersome).

Next we studied the stability of the system at the steady states for given types of interaction. As the extinction of the both species is trivial, we primarily focused on the cases

where at least one species survives, which require the following condition:

$$\alpha > 1 + \kappa \tag{32}$$

for positive κ.

We found that, as long as the above condition is satisfied, the state with both u and v extinct is linearly unstable as desired. Evaluating the Jacobian for the other two single species fixed points shows that the u nonzero, v extinct state is stable as long as $\xi_2 > 0$. Similarly, the u extinct, v nonzero state is stable as long as $\xi_1 > 0$.

A numerical exploration of the number of stable steady states in Mathematica confirmed the above analytical results. For parameter values of $\alpha = 2$ and $\kappa = 1/3$, we explored the impacts of social interaction on the system's steady state properties by varying the values of ξ_1 and ξ_2 from -0.2 to 0.2. Here, the values for ξ_1 and ξ_2 were kept relatively small to reflect the realistic case where cellular interactions from toxins or public goods are a secondary effect compared with that from nutrition. As shown in Figure 6 in the main text, the different values for ξ_1 and ξ_2 lead to different outcomes (extinction, bistable extinction, and coexistence). The axes in the plot are marginal cases (with zero eigenvalues), however, the test cases through simulations show extinction (SI Figure S12). For the figures, Figure 6C in the main text and Supporting Figure S12, interaction parameters (ξ_1 and ξ_2) were assigned 0, -0.1, or 0.1 depending on the interaction type being tested.

3 Supporting Figures

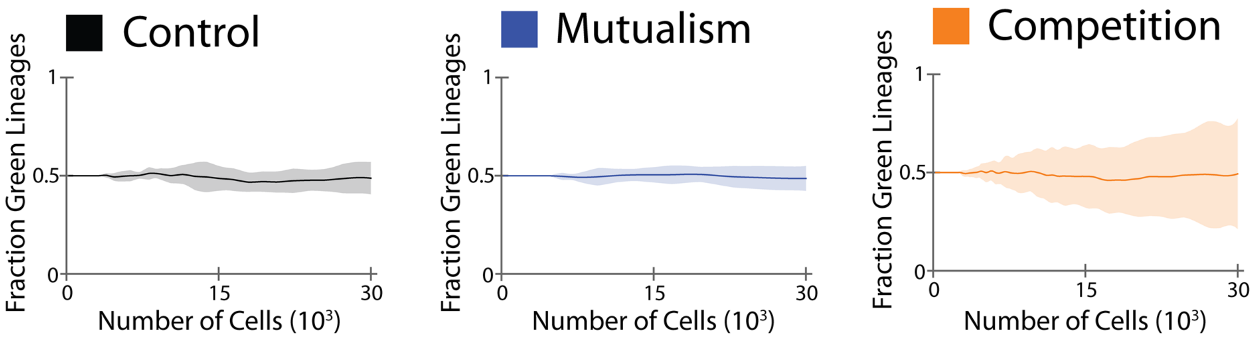

Figure S1: The fraction of green cell lineages as a function of the total cells of the communities that are neutral, mutualistic, and competing. It is a supplement to Figure 4C in the main text.

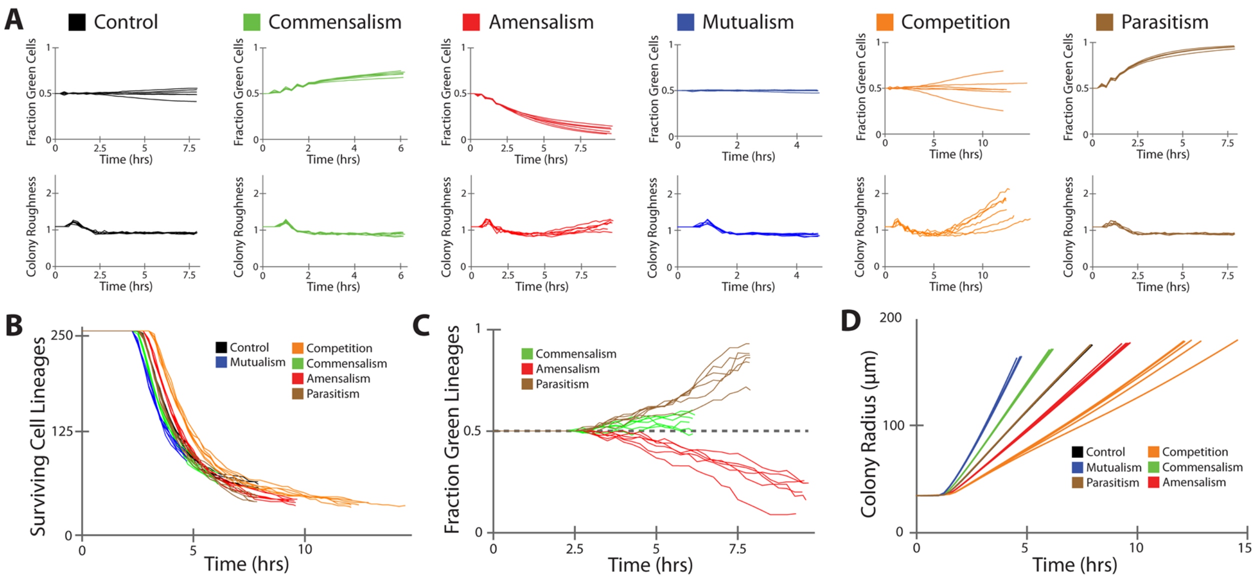

Figure S2: Statistical analysis of bacterial communities that are compared according to time evolution. Six simulation runs were performed for each interaction type in order to obtain the statistics.

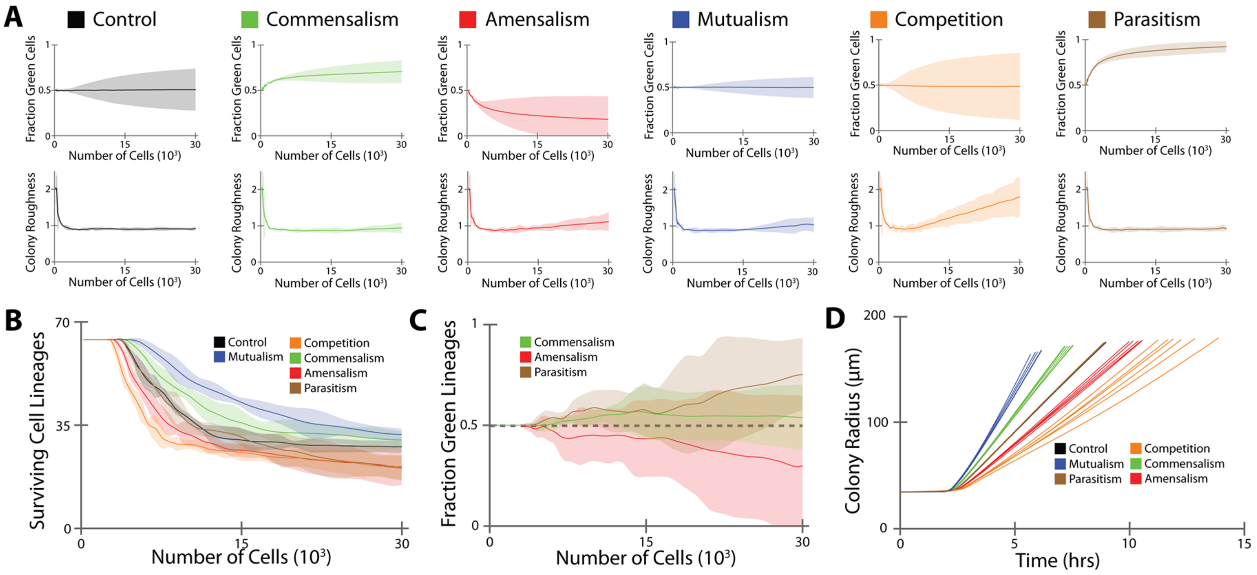

Figure S3: Statistical analysis of the role of social interactions in determining community structure for the case of a medium initial density (64 cells, 1:1 ratio).

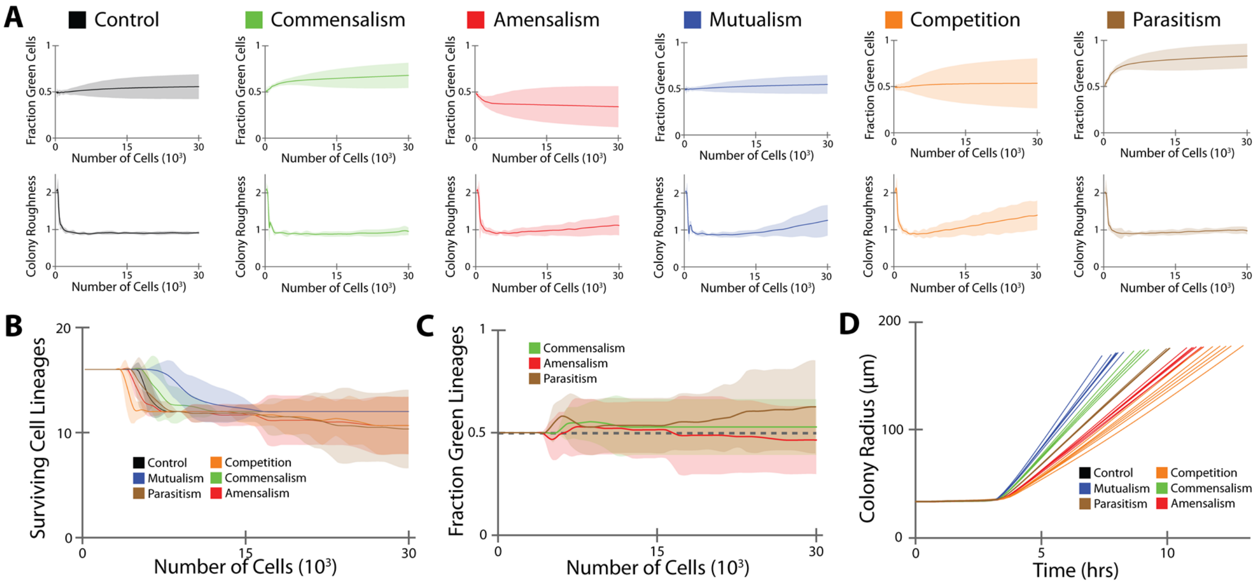

Figure S4: Statistical analysis of the role of social interactions in determining community structure for the case of a medium initial density (16 cells, 1:1 ratio).

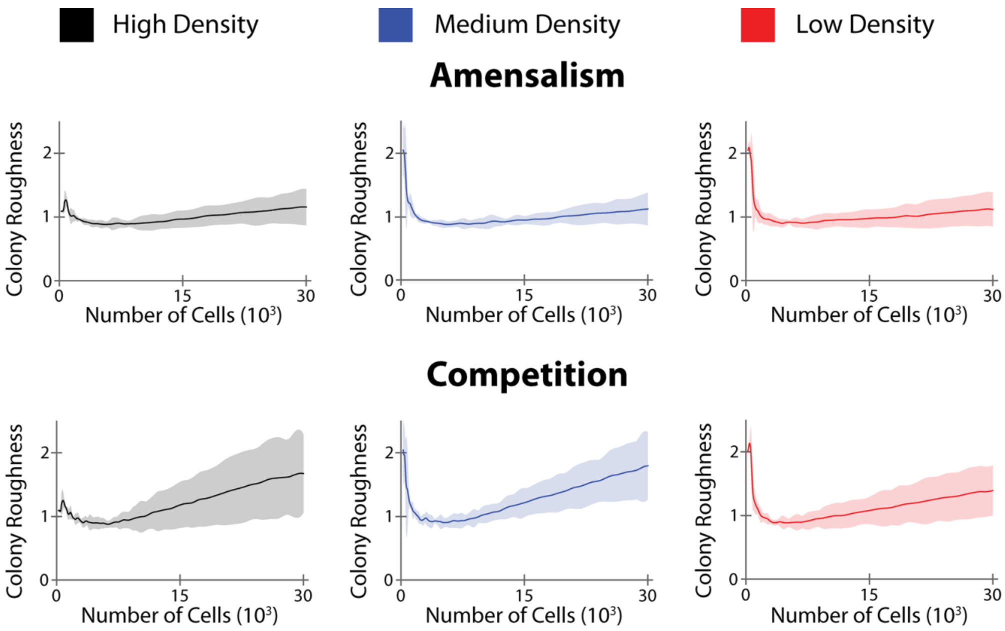

Figure S5: Changing the initial density has little effect on colony roughness for both amensalism and competition.

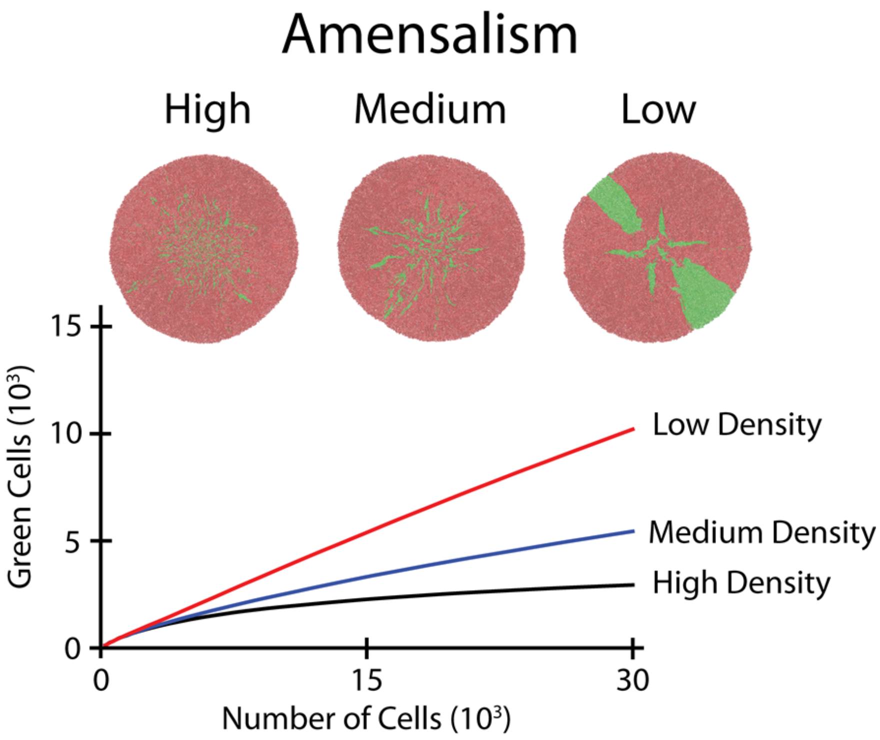

Figure S6: Representative structures emerged from amensal communities with three different initial densities: high (256 cells), medium (64 cells) and low (16 cells). As the initial cell density drops, the number of total green cells (victim species) increases, eventually resulting in coexistence.

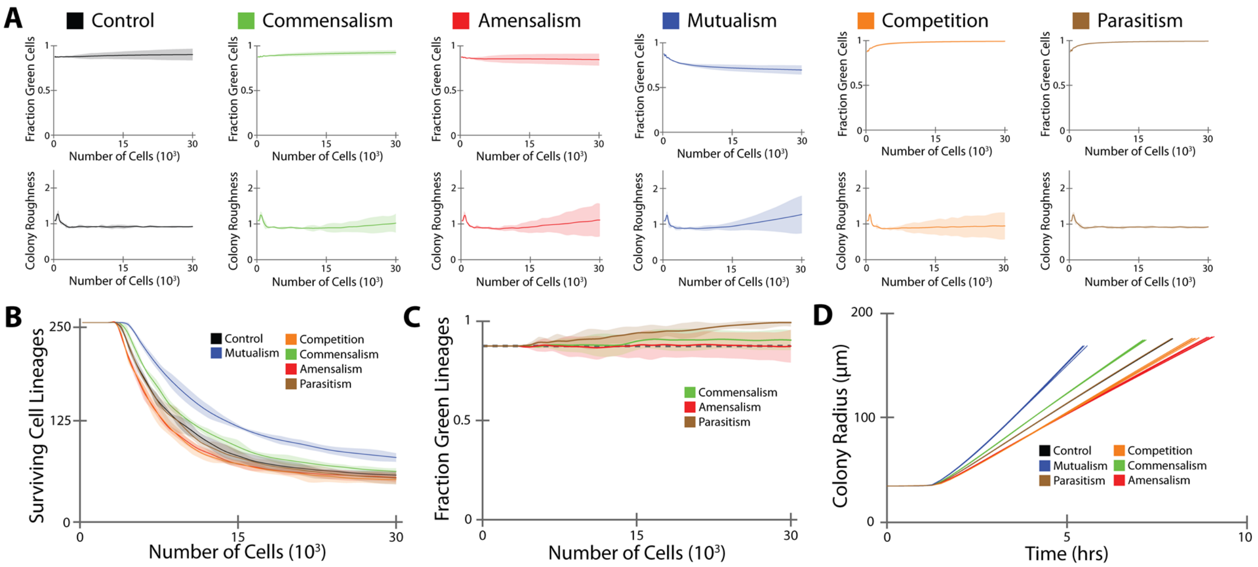

Figure S7: Statistical analysis of the role of social interactions in determining community structure for the case of a high initial density (256 cells) and a 1:7 relative abundance.

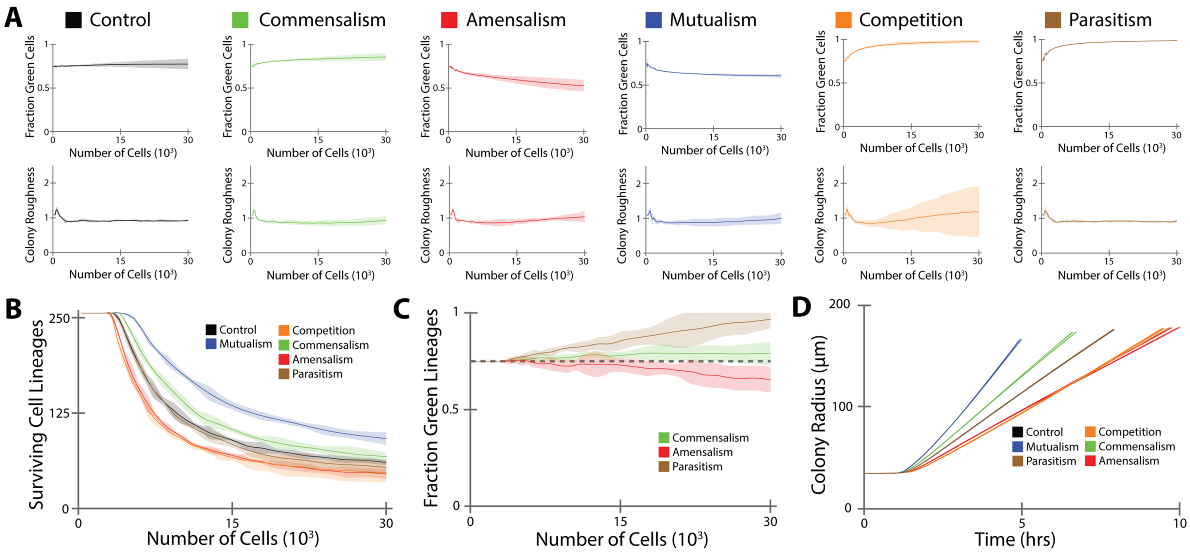

Figure S8: Statistical analysis of the role of social interactions in determining community structure for the case of a high initial density (256 cells) and a 1:3 relative abundance.

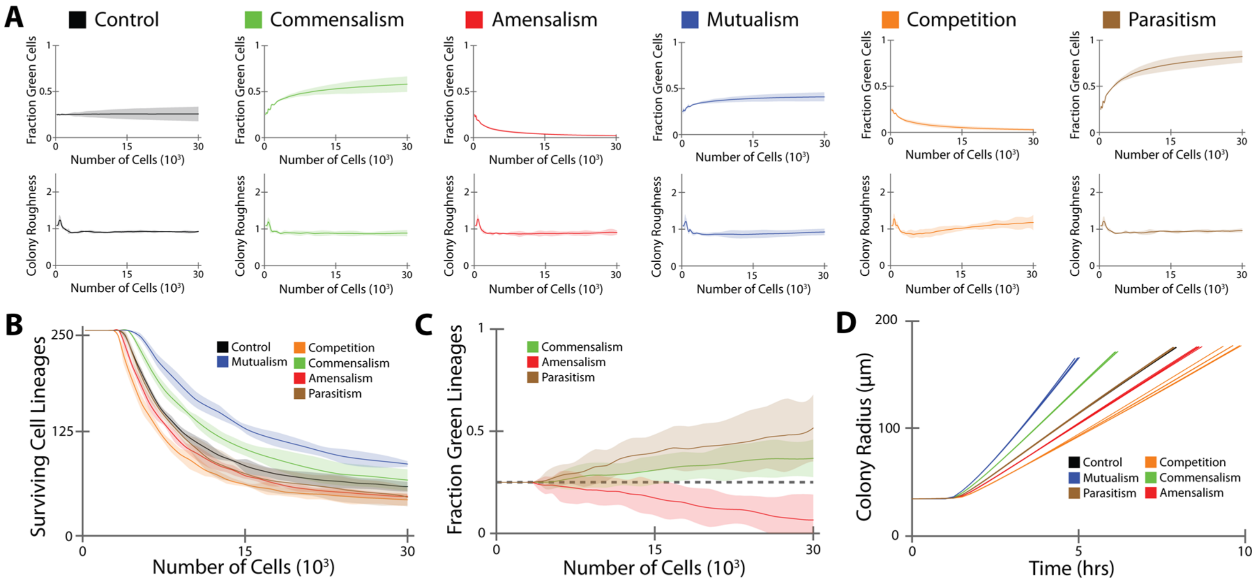

Figure S9: Statistical analysis of the role of social interactions in determining community structure for the case of a high initial density (256 cells) and a 3:1 relative abundance.

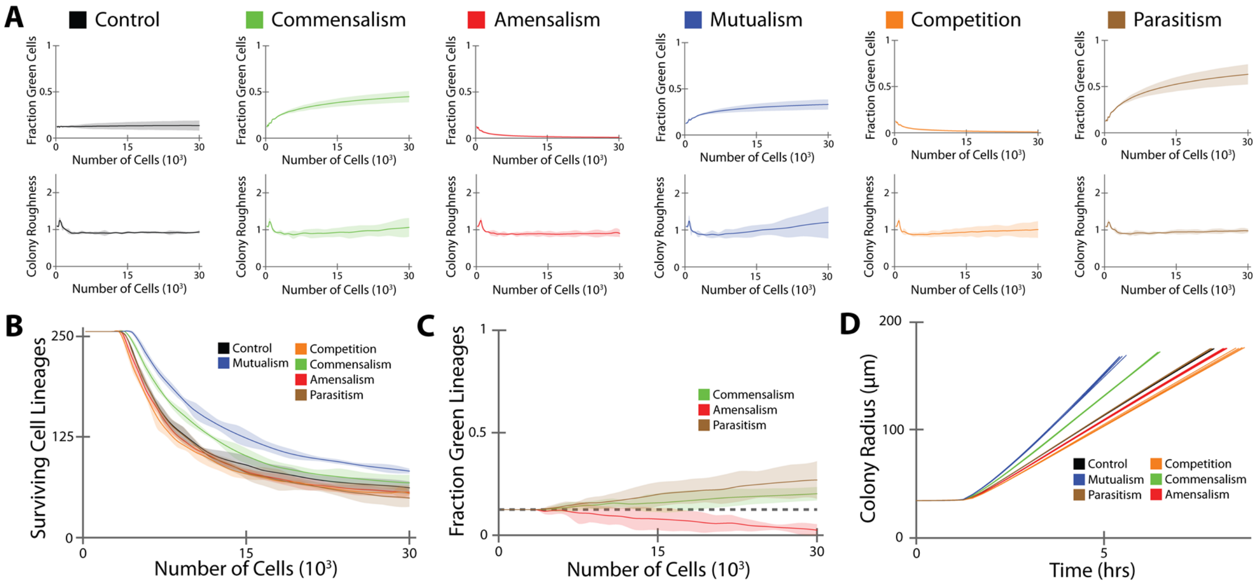

Figure S10: Statistical analysis of the role of social interactions in determining community structure for the case of a high initial density (256 cells) and a 7:1 relative abundance.

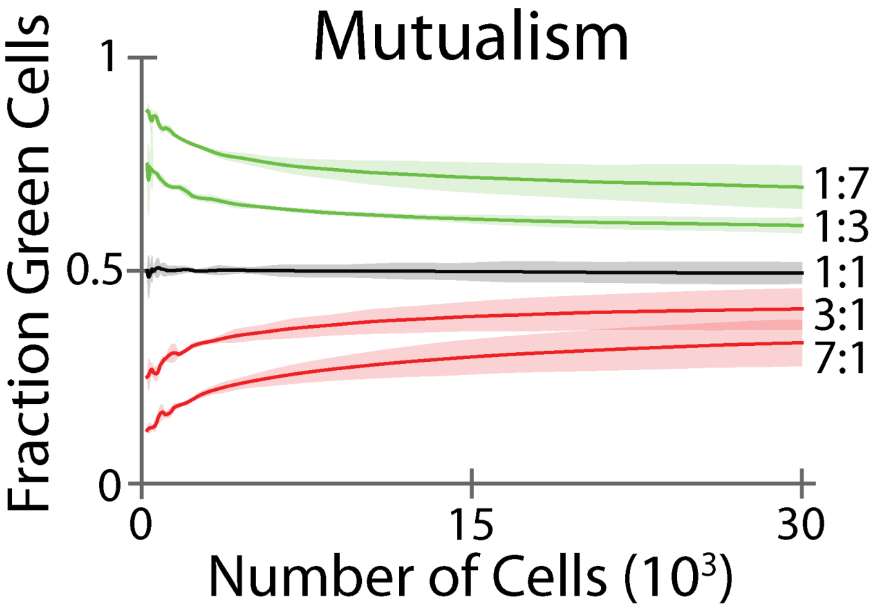

Figure S11: Fraction of green cells as a function of total cells in mutualistic communities with different initial relative abundance. Mutualism tends to decrease deviations in the relative abundance away from 1:1.

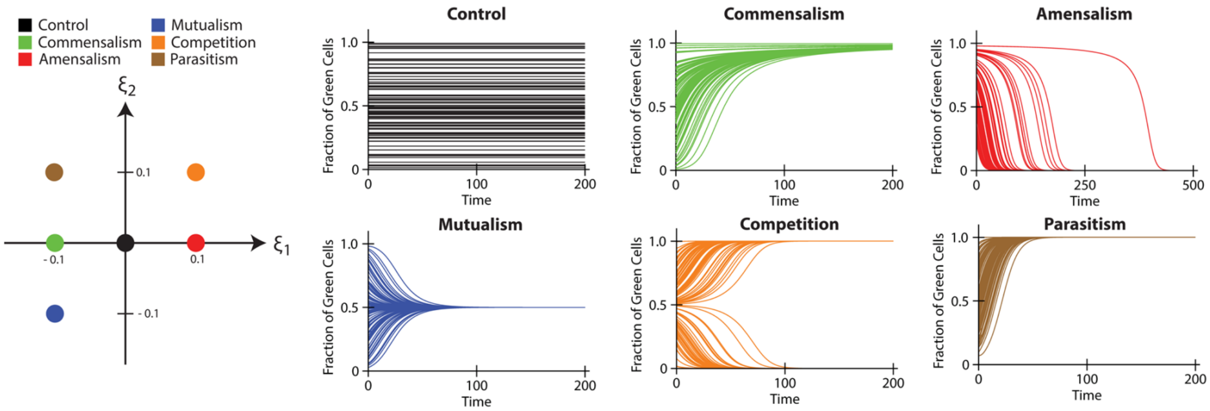

Figure S12: Simulations of well-mixed two-species communities with different social interactions. For control, the green cell fraction is determined purely by the initial conditions. For commensalism, the red cells always go extinct. For amensalism, the green cells always go extinct. For mutualism, equal species abundance is achieved. For competition, one of the species goes extinct depending on initial conditions. For parasitism, the red cells always go extinct. This figure supplements Figure 6 in the main text.

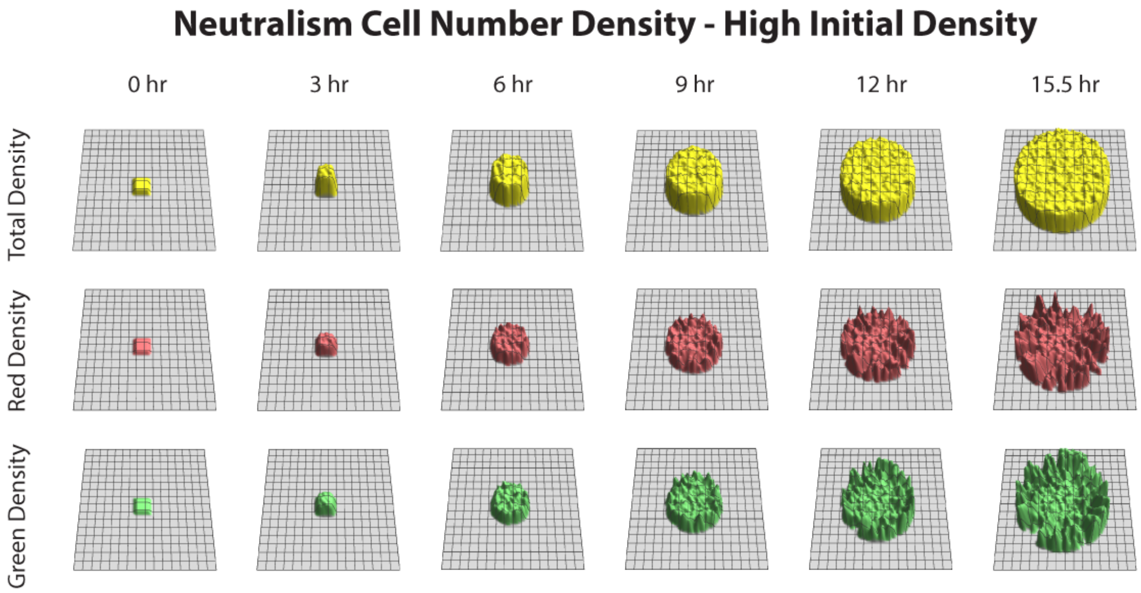

Figure S13: Time course images for cell density using the well-mixed initial conditions with high cell density and Neutralism. The total cell density (top row) shows a densely packed quasi-circular colony with a maximum height corresponding to approximately 10 cells packed in one spacial grid (5 μm by 5 μm). The values for cell density were calculated by counting the number of cells with a center of mass at a specified spatial grid point. Since cells with a center of mass at one grid point may extend into a neighboring grid, the values were averaged over nearest neighbors. The cell densities for individual species (i.e. green or red) are shown in rows 2 and 3. The mesh lines shown in the image correspond to approximately 6 spatial grids in the simulation. The boundary of the grid was chosen for display purposes; the actual simulation boundary is more distant from the expanding colony to minimize effects from the boundary conditions.

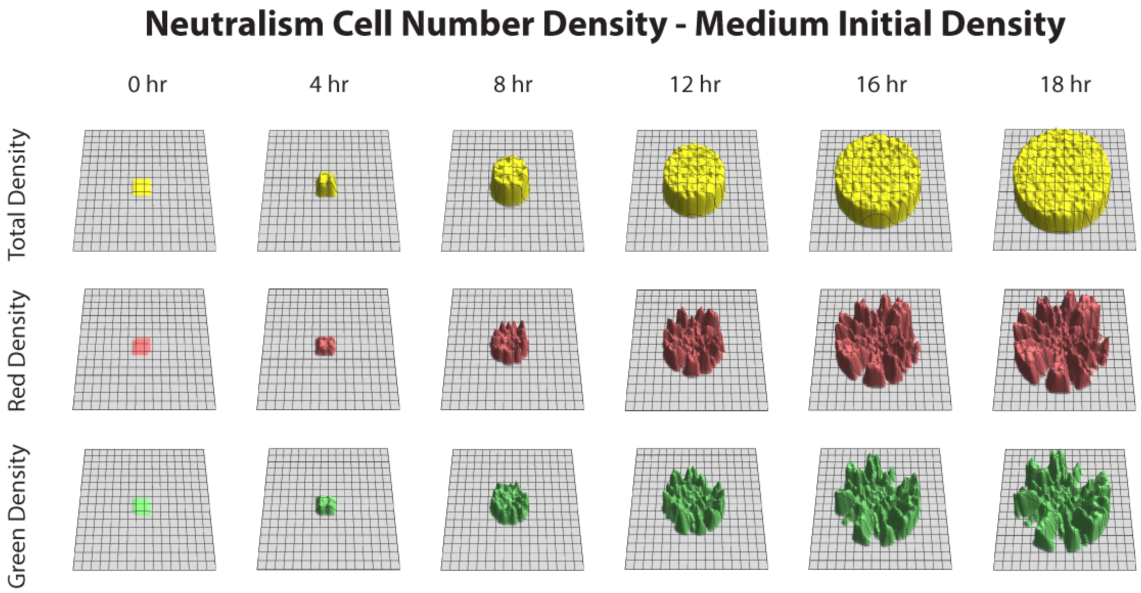

Figure S14: Time course images for cell density using the well-mixed initial conditions with medium cell density and Neutralism. The total cell density (top row) shows a densely packed quasi-circular colony with a maximum height corresponding to approximately 10 cells packed in one spacial grid (5 μm by 5 μm). The values for cell density were calculated by counting the number of cells with a center of mass at a specified spatial grid point. Since cells with a center of mass at one grid point may extend into a neighboring grid, the values were averaged over nearest neighbors. The cell densities for individual species (i.e. green or red) are shown in rows 2 and 3. The mesh lines shown in the image correspond to approximately 6 spatial grids in the simulation. The boundary of the grid was chosen for display purposes; the actual simulation boundary is more distant from the expanding colony to minimize effects from the boundary conditions.

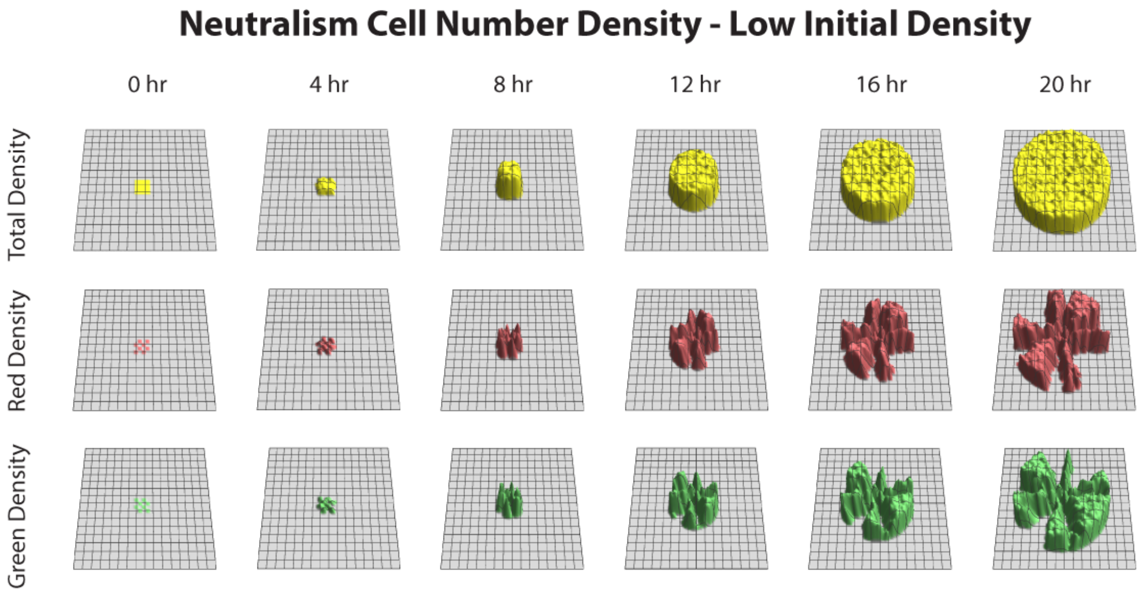

Figure S15: Time course images for cell density using the well-mixed initial conditions with low cell density and Neutralism. The total cell density (top row) shows a densely packed quasi-circular colony with a maximum height corresponding to approximately 10 cells packed in one spacial grid (5 μm by 5 μm). The values for cell density were calculated by counting the number of cells with a center of mass at a specified spatial grid point. Since cells with a center of mass at one grid point may extend into a neighboring grid, the values were averaged over nearest neighbors. The cell densities for individual species (i.e. green or red) are shown in rows 2 and 3. The mesh lines shown in the image correspond to approximately 6 spatial grids in the simulation. The boundary of the grid was chosen for display purposes; the actual simulation boundary is more distant from the expanding colony to minimize effects from the boundary conditions.

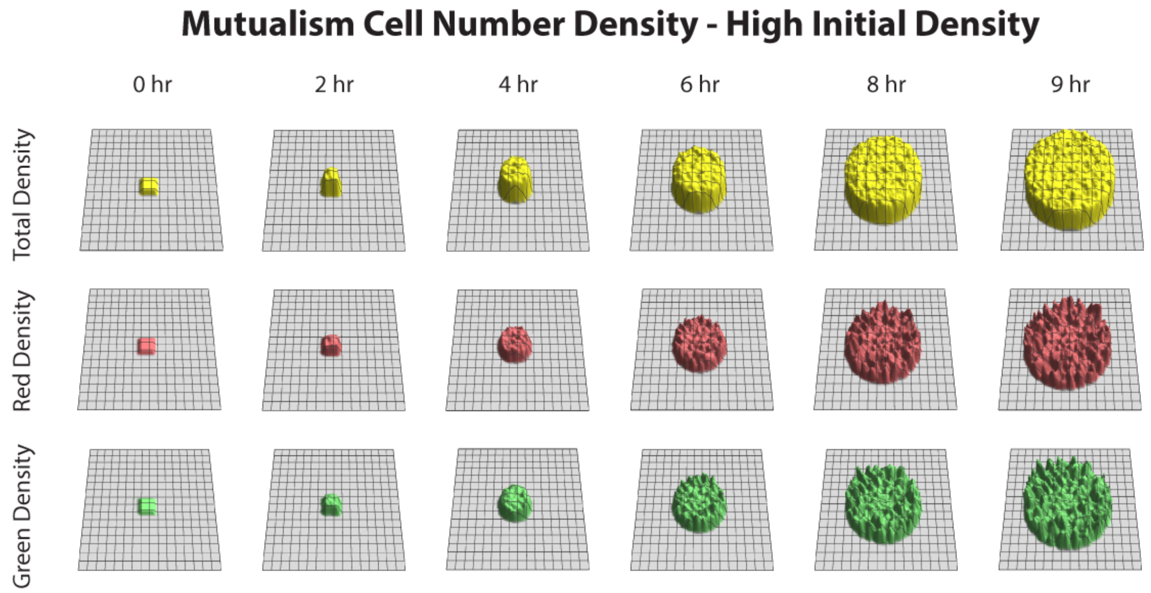

Figure S16: Time course images for cell density using the well-mixed initial conditions with high cell density and Mutualism. The total cell density (top row) shows a densely packed quasi-circular colony with a maximum height corresponding to approximately 10 cells packed in one spacial grid (5 μm by 5 μm). The values for cell density were calculated by counting the number of cells with a center of mass at a specified spatial grid point. Since cells with a center of mass at one grid point may extend into a neighboring grid, the values were averaged over nearest neighbors. The cell densities for individual species (i.e. green or red) are shown in rows 2 and 3. The mesh lines shown in the image correspond to approximately 6 spatial grids in the simulation. The boundary of the grid was chosen for display purposes; the actual simulation boundary is more distant from the expanding colony to minimize effects from the boundary conditions.

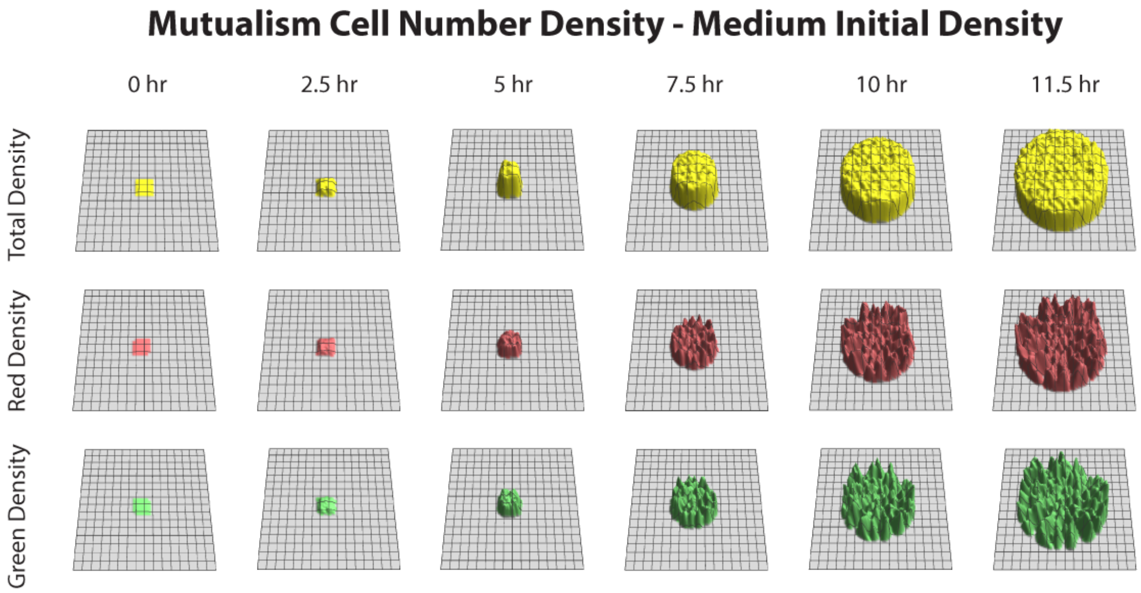

Figure S17: Time course images for cell density using the well-mixed initial conditions with medium cell density and Mutualism. The total cell density (top row) shows a densely packed quasi-circular colony with a maximum height corresponding to approximately 10 cells packed in one spacial grid (5 μm by 5 μm). The values for cell density were calculated by counting the number of cells with a center of mass at a specified spatial grid point. Since cells with a center of mass at one grid point may extend into a neighboring grid, the values were averaged over nearest neighbors. The cell densities for individual species (i.e. green or red) are shown in rows 2 and 3. The mesh lines shown in the image correspond to approximately 6 spatial grids in the simulation. The boundary of the grid was chosen for display purposes; the actual simulation boundary is more distant from the expanding colony to minimize effects from the boundary conditions.

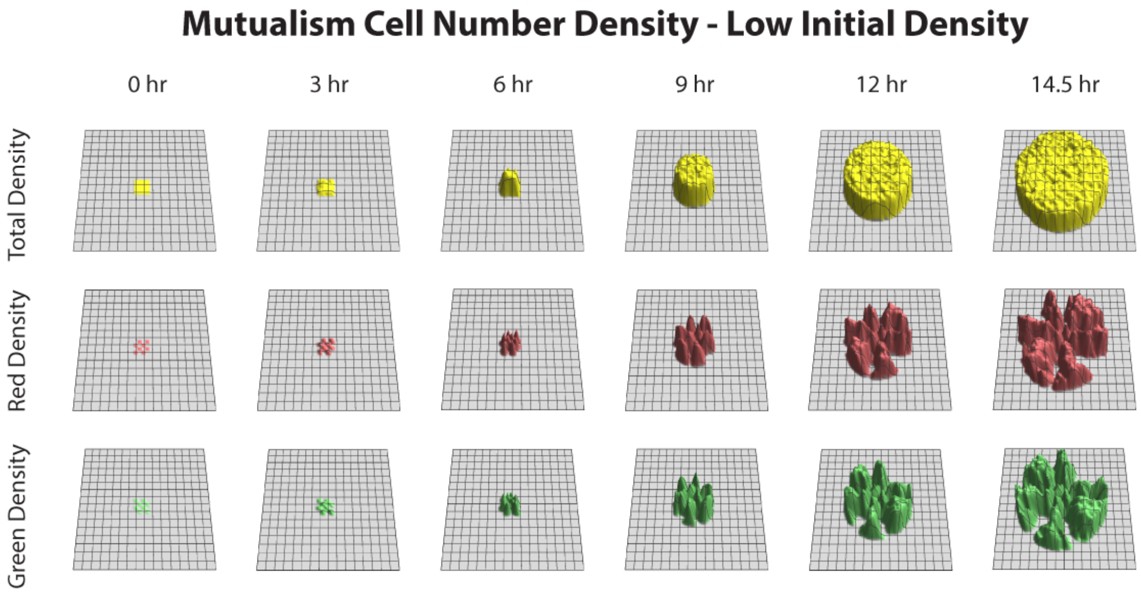

Figure S18: Time course images for cell density using the well-mixed initial conditions with low cell density and Mutualism. The total cell density (top row) shows a densely packed quasi-circular colony with a maximum height corresponding to approximately 10 cells packed in one spacial grid (5 μm by 5 μm). The values for cell density were calculated by counting the number of cells with a center of mass at a specified spatial grid point. Since cells with a center of mass at one grid point may extend into a neighboring grid, the values were averaged over nearest neighbors. The cell densities for individual species (i.e. green or red) are shown in rows 2 and 3. The mesh lines shown in the image correspond to approximately 6 spatial grids in the simulation. The boundary of the grid was chosen for display purposes; the actual simulation boundary is more distant from the expanding colony to minimize effects from the boundary conditions.

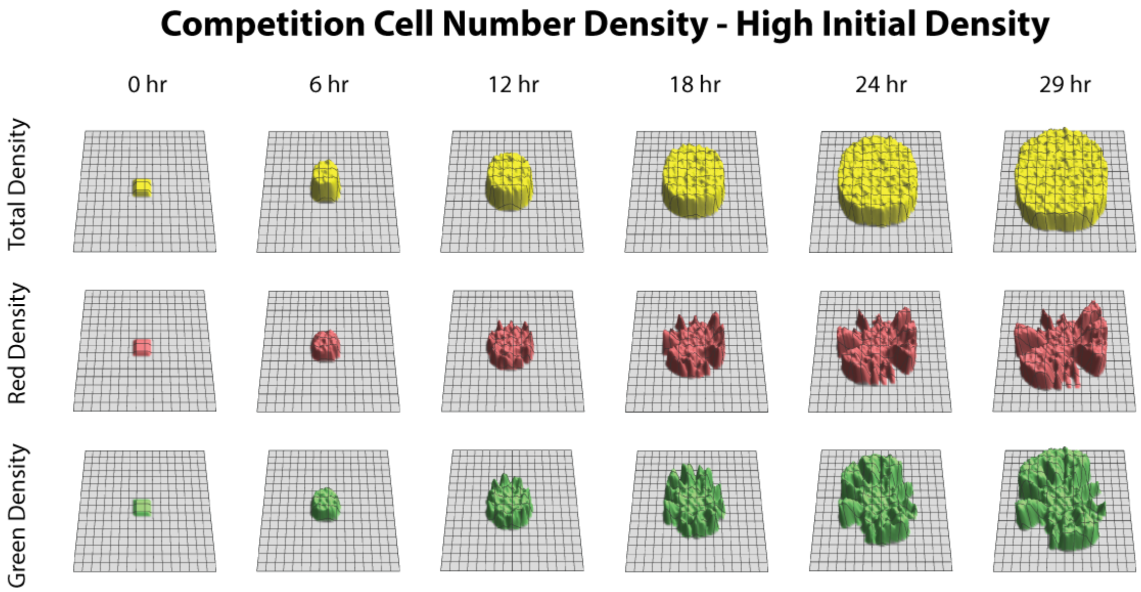

Figure S19: Time course images for cell density using the well-mixed initial conditions with high cell density and Competition. The total cell density (top row) shows a densely packed quasi-circular colony with a maximum height corresponding to approximately 10 cells packed in one spacial grid (5 μm by 5 μm). The values for cell density were calculated by counting the number of cells with a center of mass at a specified spatial grid point. Since cells with a center of mass at one grid point may extend into a neighboring grid, the values were averaged over nearest neighbors. The cell densities for individual species (i.e. green or red) are shown in rows 2 and 3. The mesh lines shown in the image correspond to approximately 6 spatial grids in the simulation. The boundary of the grid was chosen for display purposes; the actual simulation boundary is more distant from the expanding colony to minimize effects from the boundary conditions.

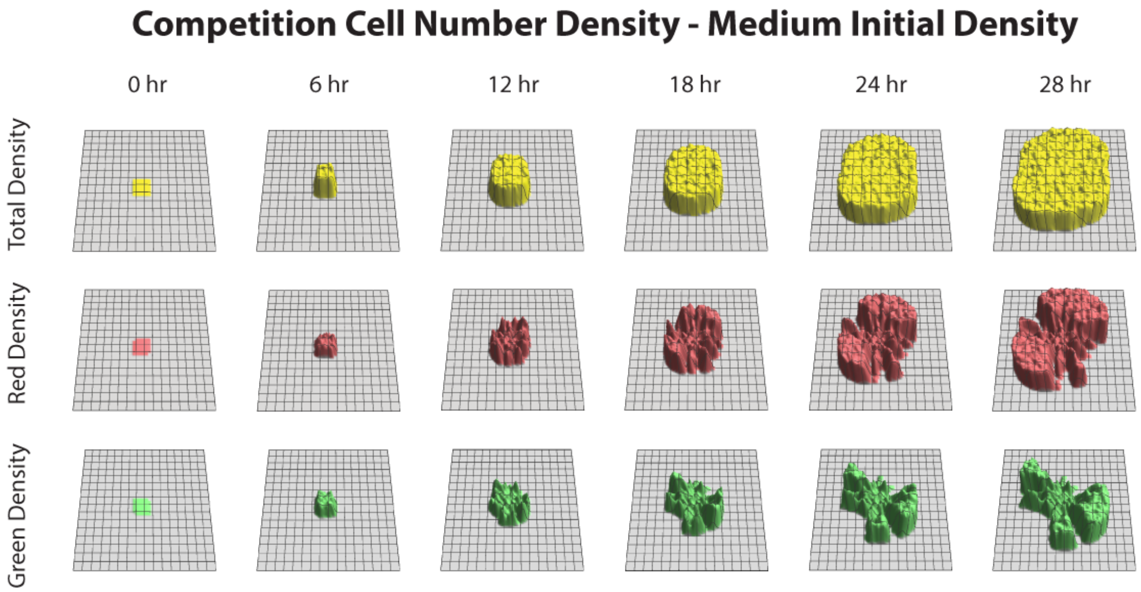

Figure S20: Time course images for cell density using the well-mixed initial conditions with medium cell density and Competition. The total cell density (top row) shows a densely packed quasi-circular colony with a maximum height corresponding to approximately 10 cells packed in one spacial grid (5 μm by 5 μm). The values for cell density were calculated by counting the number of cells with a center of mass at a specified spatial grid point. Since cells with a center of mass at one grid point may extend into a neighboring grid, the values were averaged over nearest neighbors. The cell densities for individual species (i.e. green or red) are shown in rows 2 and 3. The mesh lines shown in the image correspond to approximately 6 spatial grids in the simulation. The boundary of the grid was chosen for display purposes; the actual simulation boundary is more distant from the expanding colony to minimize effects from the boundary conditions.

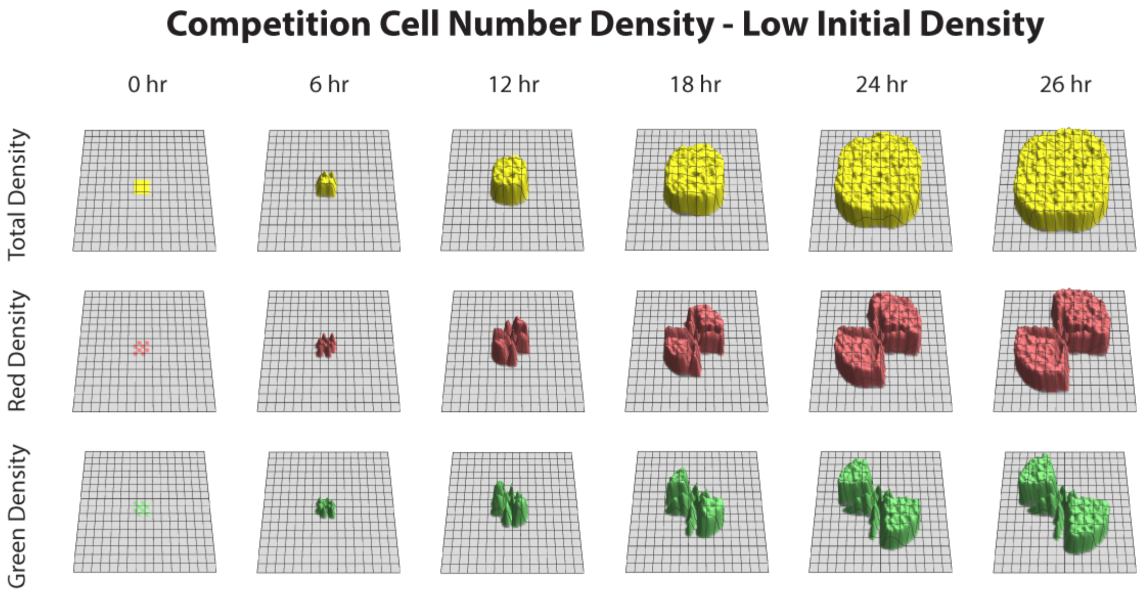

Figure S21: Time course images for cell density using the well-mixed initial conditions with low cell density and Competition. The total cell density (top row) shows a densely packed quasi-circular colony with a maximum height corresponding to approximately 10 cells packed in one spacial grid (5 μm by 5 μm). The values for cell density were calculated by counting the number of cells with a center of mass at a specified spatial grid point. Since cells with a center of mass at one grid point may extend into a neighboring grid, the values were averaged over nearest neighbors. The cell densities for individual species (i.e. green or red) are shown in rows 2 and 3. The mesh lines shown in the image correspond to approximately 6 spatial grids in the simulation. The boundary of the grid was chosen for display purposes; the actual simulation boundary is more distant from the expanding colony to minimize effects from the boundary conditions.

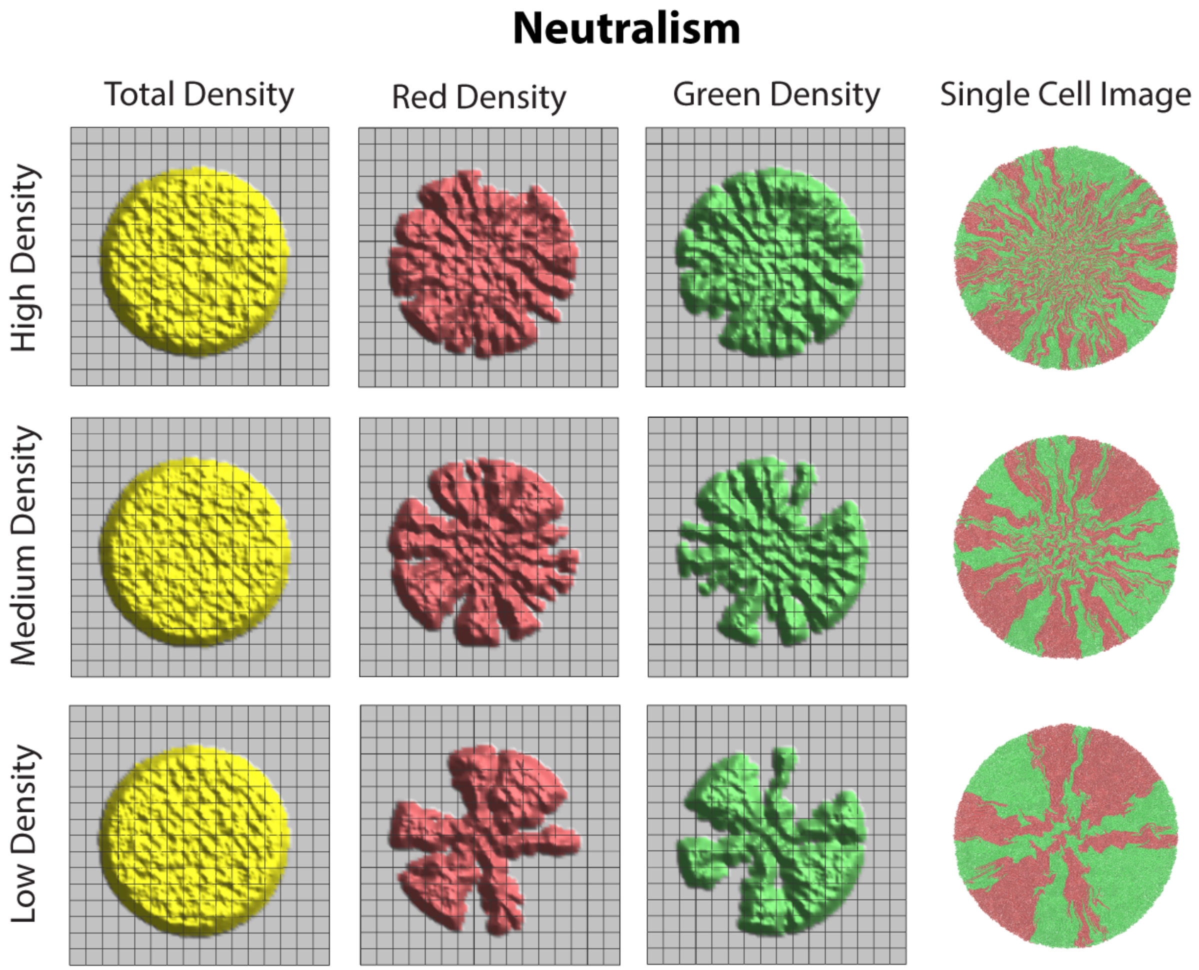

Figure S22: Comparison of the cell number density plots and the single cell image for a top down view of the final state for a simulation with well-mixed initial conditions and Neutralism. The two-dimensional nature of the simulations allows the local density to be inferred by considering the color of the single cells in the images of the far right column.

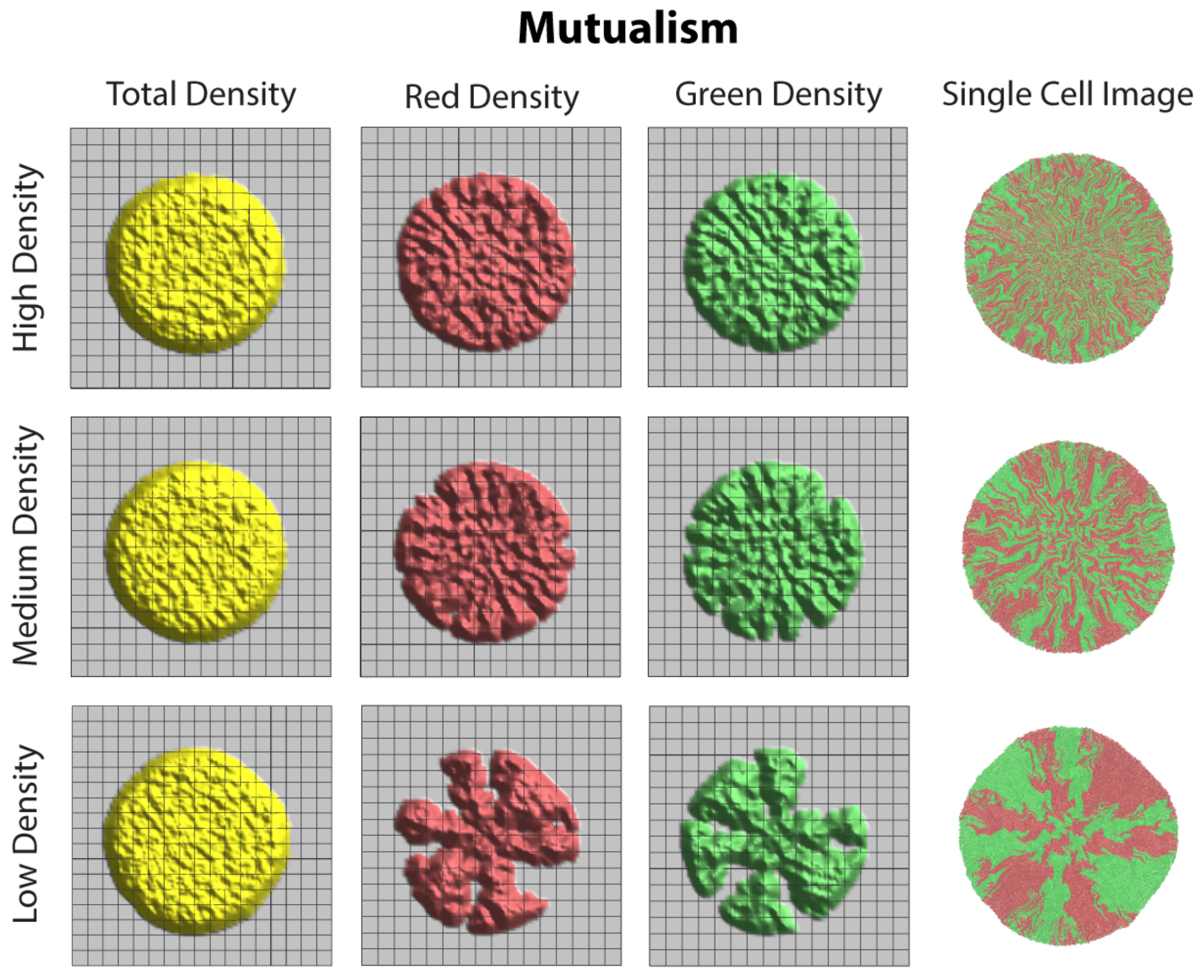

Figure S23: Comparison of the cell number density plots and the single cell image for a top down view of the final state for a simulation with well-mixed initial conditions and Mutualism. The two-dimensional nature of the simulations allows the local density to be inferred by considering the color of the single cells in the images of the far right column.

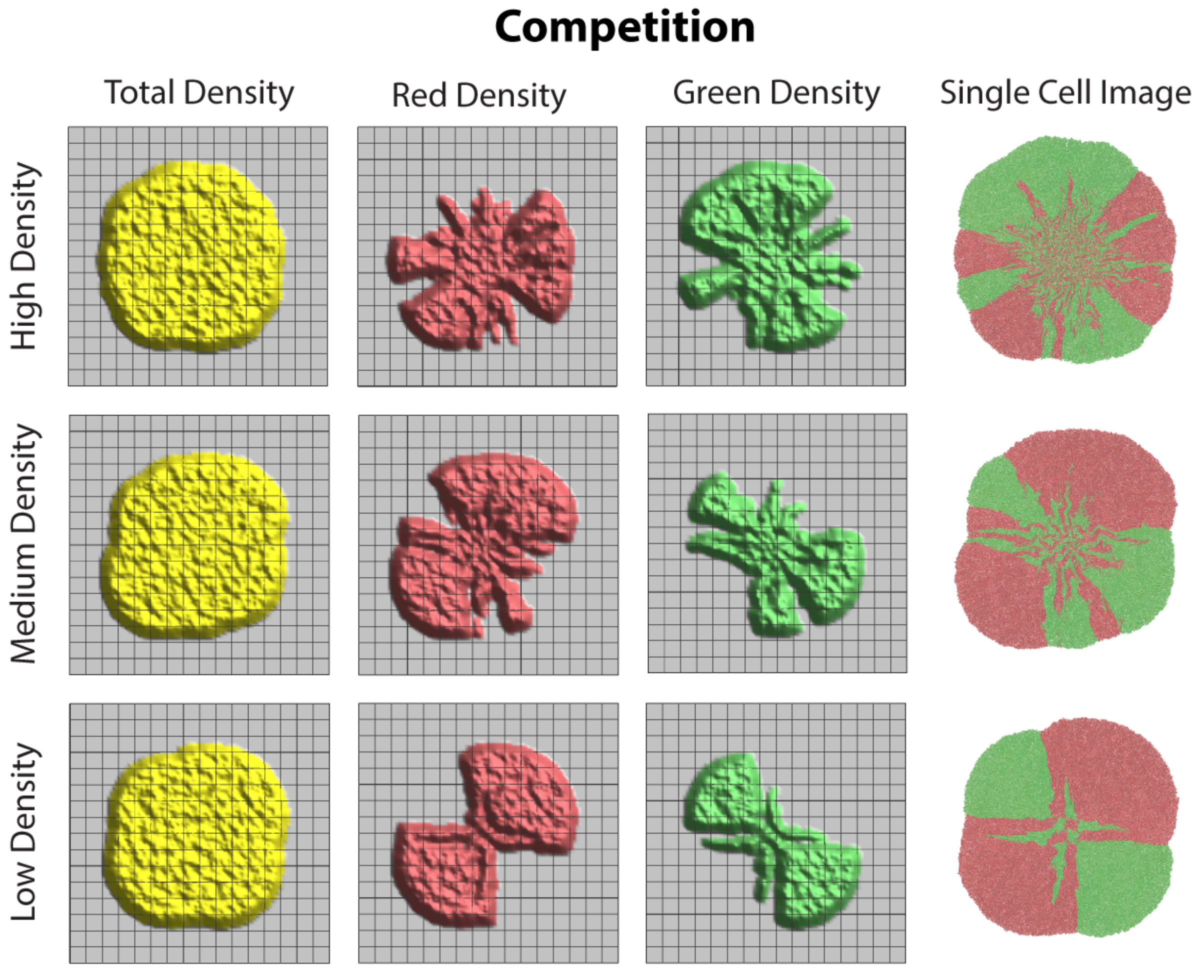

Figure S24: Comparison of the cell number density plots and the single cell image for a top down view of the final state for a simulation with well-mixed initial conditions and Competition. The two-dimensional nature of the simulations allows the local density to be inferred by considering the color of the single cells in the images of the far right column.

4 Supporting Tables

Table S3: Run statistics for the case with an equal initial relative abundance and a medium initial density.

	Control	Commensalism	Amensalism	Competition	Mutualism	Parasitism
Green Fraction	0.51 ± 0.12	0.71 ± 0.06	0.18 ± 0.13	0.49 ± 0.18	0.50 ± 0.06	0.92 ± 0.03
Roughness	0.92 ± 0.02	0.94 ± 0.07	1.11 ± 0.13	1.79 ± 0.26	1.02 ± 0.11	0.92 ± 0.03
Lineages	27.67 ± 1.03	30.00 ± 1.41	21.00 ± 3.10	20.50 ± 2.17	31.83 ± 0.98	20.50 ± 2.07
Green Lineages	14.17 ± 1.94	16.17 ± 2.64	6.67 ± 4.76	10.00 ± 3.35	16.00 ± 2.28	15.33 ± 1.51
Sectors	15.17 ± 3.87	16.50 ± 4.76	4.00 ± 2.53	5.17 ± 2.64	24.33 ± 5.57	1.67 ± 0.82

Table S4: Run statistics for the case with an equal initial relative abundance and a low initial density.

	Control	Commensalism	Amensalism	Competition	Mutualism	Parasitism
Green Fraction	0.56 ± 0.07	0.68 ± 0.07	0.34 ± 0.11	0.54 ± 0.13	0.55 ± 0.05	0.83 ± 0.07
Roughness	0.91 ± 0.02	0.95 ± 0.05	1.11 ± 0.13	1.39 ± 0.20	1.26 ± 0.21	0.98 ± 0.05
Lineages	12.00 ± 0.00	12.00 ± 0.00	10.67 ± 1.37	10.67 ± 1.37	12.00 ± 0.00	10.33 ± 1.86
Green Lineages	6.33 ± 0.82	6.33 ± 0.82	5.00 ± 1.26	5.33 ± 1.75	6.33 ± 0.82	6.33 ± 0.82
Sectors	7.83 ± 3.82	6.83 ± 2.99	4.33 ± 2.34	3.67 ± 1.51	12.00 ± 3.41	3.33 ± 1.51

Table S5: Run statistics for the case with a 1:7 initial relative abundance and a high initial density.

	Control	Commensalism	Amensalism	Competition	Mutualism	Parasitism
Green Fraction	0.90 ± 0.03	0.92 ± 0.01	0.85 ± 0.03	0.99 ± 0.00	0.70 ± 0.03	0.99 ± 0.00
Roughness	0.92 ± 0.01	1.02 ± 0.13	1.11 ± 0.23	0.94 ± 0.19	1.27 ± 0.26	0.92 ± 0.01
Lineages	60.20 ± 2.39	65.00 ± 2.00	57.20 ± 2.95	54.40 ± 2.61	82.80 ± 2.77	56.40 ± 4.10
Green Lineages	54.20 ± 1.30	58.80 ± 2.59	50.00 ± 3.08	53.60 ± 2.70	70.60 ± 3.05	56.00 ± 4.12
Sectors	5.80 ± 2.77	4.40 ± 2.30	8.60 ± 0.89	1.00 ± 0.00	20.40 ± 3.51	1.00 ± 0.00

Table S6: Run statistics the case with a 1:3 initial relative abundance and a high density.

	Control	Commensalism	Amensalism	Competition	Mutualism	Parasitism
Green Fraction	0.77 ± 0.03	0.86 ± 0.02	0.53 ± 0.03	0.98 ± 0.01	0.61 ± 0.01	0.99 ± 0.00
Roughness	0.93 ± 0.01	0.93 ± 0.06	1.04 ± 0.09	1.17 ± 0.35	1.00 ± 0.08	0.92 ± 0.02
Lineages	60.40 ± 1.52	68.00 ± 3.00	45.40 ± 3.44	47.00 ± 6.36	91.80 ± 4.38	53.80 ± 4.21
Green Lineages	46.00 ± 2.83	53.80 ± 3.42	29.80 ± 3.11	45.00 ± 5.29	65.80 ± 3.70	52.00 ± 3.94
Sectors	16.80 ± 1.48	7.20 ± 4.15	9.80 ± 2.77	1.20 ± 0.45	29.00 ± 4.30	1.00 ± 0.00

Table S7: Run statistics for a 3:1 initial relative abundance and a high initial density.

	Control	Commensalism	Amensalism	Competition	Mutualism	Parasitism
Green Fraction	0.26 ± 0.04	0.58 ± 0.04	0.02 ± 0.00	0.03 ± 0.01	0.41 ± 0.02	0.82 ± 0.03
Roughness	0.92 ± 0.01	0.89 ± 0.04	0.90 ± 0.03	1.17 ± 0.10	0.92 ± 0.04	0.96 ± 0.03
Lineages	61.20 ± 3.03	69.40 ± 6.69	49.20 ± 1.64	44.80 ± 3.42	89.40 ± 1.82	48.20 ± 5.76
Green Lineages	17.00 ± 2.35	25.40 ± 3.29	3.20 ± 2.95	2.20 ± 1.30	27.60 ± 2.88	24.60 ± 3.21
Sectors	15.20 ± 5.40	21.40 ± 4.39	1.00 ± 0.00	1.40 ± 0.55	29.60 ± 4.62	5.40 ± 0.89

Table S8: Run statistics for a 7:1 initial relative abundance and a high initial density.

	Control	Commensalism	Amensalism	Competition	Mutualism	Parasitism
Green Fraction	0.14 ± 0.03	0.45 ± 0.03	0.01 ± 0.00	0.01 ± 0.00	0.33 ± 0.03	0.63 ± 0.05
Roughness	0.93 ± 0.03	1.06 ± 0.13	0.90 ± 0.05	1.00 ± 0.11	1.20 ± 0.22	0.97 ± 0.03
Lineages	61.80 ± 3.96	68.00 ± 4.95	56.40 ± 3.97	54.80 ± 1.48	82.60 ± 1.52	49.20 ± 5.45
Green Lineages	9.40 ± 2.07	13.80 ± 1.92	1.40 ± 0.89	1.40 ± 1.52	13.80 ± 2.17	13.20 ± 2.28
Sectors	9.40 ± 2.30	18.60 ± 5.50	1.00 ± 0.00	1.00 ± 0.00	24.20 ± 5.97	6.80 ± 1.64

Table S9: Run statistics for communities simulated using the well-mixed model. The mean and standard deviation of the fraction of green cells were obtained using 1000 runs of simulations with random initial conditions (each of the species concentrations is between 0 and 1 and nutrition is set to be 1).

	Control	Competition	Mutualism
Green Fraction	0.50 ± 0.24	0.48 ± 0.50	0.50 ± 0.00

References

(S1) Farrell, F. D. C., Hallatschek, O., Marenduzzo, D., and Waclaw, B. (2013) Mechanically Driven Growth of Quasi-Two-Dimensional Microbial Colonies. *Phys. Rev. Lett. 111*, 168101.

(S2) Pöschel, T. and Schwager, T., *Computational Granular Dynamics*; Springer: Berlin, 2005.

(S3) Monod, J. (1949) The Growth of Bacterial Cultures. *Annu. Rev. Microbiol. 3*, 371–394.

(S4) Müller, M. J. I., Neugeboren, B. I., Nelson, D. R., and Murray, A. W. (2014) Genetic drift opposes mutualism during spatial population expansion. *Proc. Natl. Acad. Sci. U.S.A. 111*, 1037–1042.
